# Heart failure-induced atrial remodelling promotes electrical and conduction alternans

**DOI:** 10.1371/journal.pcbi.1008048

**Published:** 2020-07-13

**Authors:** Na Zhao, Qince Li, Kevin Zhang, Kuanquan Wang, Runnan He, Yongfeng Yuan, Henggui Zhang

**Affiliations:** 1 School of Computer Science and Technology, Harbin Institute of Technology, Harbin, China; 2 Peng Cheng Laboratory, Shenzhen, China; 3 School of Medicine, Imperial College of London, United Kingdom; 4 School of Physics & Astronomy, The University of Manchester, Manchester, United Kingdom; 5 Key Laboratory of Medical Electrophysiology of Ministry of Education and Medical Electrophysiological Key Laboratory of Sichuan Province, Institute of Cardiovascular Research, Southwest Medical University, Luzhou, China; University of Michigan, UNITED STATES

## Abstract

Heart failure (HF) is associated with an increased propensity for atrial fibrillation (AF), causing higher mortality than AF or HF alone. It is hypothesized that HF-induced remodelling of atrial cellular and tissue properties promotes the genesis of atrial action potential (AP) alternans and conduction alternans that perpetuate AF. However, the mechanism underlying the increased susceptibility to atrial alternans in HF remains incompletely elucidated. In this study, we investigated the effects of how HF-induced atrial cellular electrophysiological (with prolonged AP duration) and tissue structural (reduced cell-to-cell coupling caused by atrial fibrosis) remodelling can have an effect on the generation of atrial AP alternans and their conduction at the cellular and one-dimensional (1D) tissue levels. Simulation results showed that HF-induced atrial electrical remodelling prolonged AP duration, which was accompanied by an increased sarcoplasmic reticulum (SR) Ca^2+^ content and Ca^2+^ transient amplitude. Further analysis demonstrated that HF-induced atrial electrical remodelling increased susceptibility to atrial alternans mainly due to the increased sarcoplasmic reticulum Ca^2+^-ATPase (SERCA) Ca^2+^ reuptake, modulated by increased phospholamban (PLB) phosphorylation, and the decreased transient outward K^+^ current (*I*_to_). The underlying mechanism has been suggested that the increased SR Ca^2+^ content and prolonged AP did not fully recover to their previous levels at the end of diastole, resulting in a smaller SR Ca^2+^ release and AP in the next beat. These produced Ca^2+^ transient alternans and AP alternans, and further caused AP alternans and Ca^2+^ transient alternans through Ca^2+^→AP coupling and AP→Ca^2+^ coupling, respectively. Simulation of a 1D tissue model showed that the combined action of HF-induced ion channel remodelling and a decrease in cell-to-cell coupling due to fibrosis increased the heart tissue’s susceptibility to the formation of spatially discordant alternans, resulting in an increased functional AP propagation dispersion, which is pro-arrhythmic. These findings provide insights into how HF promotes atrial arrhythmia in association with atrial alternans.

## Author summary

Atrial Fibrillation (AF) is the most common arrhythmia in adults, especially in the elderly, with the increased incidence of stroke being a major complication that increases morbidity and mortality. The occurrence of AF is often accompanied by heart failure (HF). AF and HF are also known to have the bidirectional relationship that AF worsens HF and HF promotes AF. HF can induce atrial remodelling, including electrical remodelling, atrial fibrosis, stretch and dilatation, and oxidative stress, in which many factors are associated with arrhythmogenic atrial alternans. HF-induced atrial remodelling varies during various stages and complications of HF, but possible mechanisms underlying their pro-susceptibility to alternans have not been completely elucidated. In this study, we investigated the effects of HF-induced atrial remodelling with prolonged action potential duration (APD) and decreased cell-to-cell coupling on susceptibility to atrial alternans. Simulation results showed that HF-induced an increase in sarcoplasmic reticulum Ca^2+^-ATPase (SERCA) Ca^2+^ reuptake caused by increased phospholamban phosphorylation and a decrease in transient outward K^+^ current played significant roles in the genesis of Ca^2+^ transient alternans and action potential alternans at the single-cell level. The HF-induced decline of cell-to-cell coupling and APD prolongation promoted the genesis of spatially discordant alternans in atrial tissue. This provides insights into how HF facilitates atrial arrhythmia in relation to atrial alternans.

## Introduction

Atrial fibrillation (AF) and heart failure (HF) are common cardiovascular diseases that frequently coexist with each other, resulting in higher mortality than AF or HF alone [1]. For example, over one-third of patients with AF in the Framingham Heart Study had HF, and more than half of patients with HF also had AF [2]. This is because HF shares many similar risk factors (e.g. advanced age, coronary disease, hypertension, diabetes mellitus, sleep apnoea, etc.) and pathophysiology to AF such that HF and AF can induce and facilitate each other [3–6]. In particular, HF-induced atrial fibrosis, electrical remodelling, stretch and dilatation, and oxidative stress promote AF [3,7]. However, its exact mechanism remains to be completely elucidated.

HF may exhibit inconsistent pathological remodelling of the atria due to the various stages and complications of cardiac disease [8,9], as demonstrated by various techniques and experimental conditions [6]. For example, some studies reported shortened action potential duration (APD) [10,11] and reduced atrial cellular effective refractory periods (ERPs) [12] in HF, which had significant roles in promoting and maintaining AF due to the shortened wavelength [13–16], whereas the study by Molina *et al*. showed no significant difference in atrial APD between control and HF patients [17]. Even more confoundingly, other studies have reported prolonged APD [18–21] and increased atrial ERP [22] in HF.

When HF was present alongside prolonged atrial APD, experimental data obtained from patients and dogs indicated an increased propensity for AF [18,19,21,22]. This appears to be associated with atrial ion channel remodelling, Ca^2+^ handling abnormalities and structural remodelling. Previous studies have found that atrial action potential (AP) alternans (beat-to-beat alterations in APs) preceded AF episodes, indicating that atrial alternans plays an important role in promoting AF [23–25]. In the atria, many factors have been shown to be associated with arrhythmogenic atrial alternans, such as mechanical stretch, myocardial infarction, hypertension, and hypertrophy [26–29]. In addition, many aspects of HF-induced atrial remodelling have been identified experimentally to also be related to atrial alternans. These include a prolonged atrial APD, a reduced L-type Ca^2+^ current (*I*_Ca_), an increased sarcoplasmic reticulum (SR) Ca^2+^ load, loss of t-tubules, decreased connexin expression and disrupted cell-to-cell coupling. Prolonged atrial APD caused by HF leads to a shortened diastolic interval (DI), resulting in an increased slope of the APD restitution curve. Some studies have demonstrated that APD alternans can be predicted by an APD restitution slope > 1 [30–33]. The reduced *I*_Ca_ may induce an increased susceptibility to Ca^2+^ alternans via a mechanism in which most ryanodine receptors (RyR2) are activated by Ca^2+^ waves that occur above a certain SR Ca^2+^ content threshold level; and an increased Ca^2+^ efflux with large Ca^2+^ transient causes a SR Ca^2+^ content to go below the threshold level, leading to a smaller Ca^2+^ transient in the next cycle [34]. In both experimental and simulation studies, SR Ca^2+^ load has been demonstrated to play a significant role in Ca^2+^ alternans [23,34–36]. In addition, the loss of t-tubules in atrial myocytes has been found in HF animal models [37], which may also contribute to the genesis of Ca^2+^ alternans [38]. Furthermore, decreased connexin expression and disrupted cell-to-cell coupling due to increased fibrosis increases tissue heterogeneity which may facilitate the development of spatially discordant alternans [39]. Though various atrial remodelling instances induced by HF have been identified and may vary at different stages, their pro-susceptibility to alternans are not completely understood yet.

This study focuses on HF with a prolonged APD and investigates the effects of atrial remodelling on the genesis of cardiac alternans based on short-term experimental data gathered from dogs who have had HF induced by no more than 5 weeks of ventricular tachypacing [18,19,40]. It aims to address how HF-induced atrial remodelling can increase susceptibility to alternans. We first modified the updated canine atrial cell model by Ramirez *et al*. [41] (the RNC model) to incorporate the effects of short-term HF-induced atrial remodelling. Then we sought to (i) illustrate the effects of this HF-induced atrial remodelling on atrial myocyte action potentials and Ca^2+^ transient; (ii) assess whether HF-induced atrial remodelling promotes susceptibility to atrial myocyte alternans and the mechanism by which this might occur; and (iii) investigate spatial alternans at the one-dimensional (1D) tissue level under the HF condition.

## Methods

### Single basal atrial cell model

This study was designed to assess the effects of HF-induced electrical remodelling on the genesis of atrial alternans, which was in association with a prolonged APD observed in canine atria with short-term HF [18,19]. Such effect was evaluated by using the canine atrial cell model developed by Ramirez *et al*. [41], with the incorporation of an updated Na/Ca exchanger (NCX) current (*I*_NCX_) formulation developed by Weber *et al*. [42] to describe more detailed regulation of the current via intracellular Ca^2+^ concentration. The parameters of the Weber *I*_NCX_ model were derived from experimental data of mouse myocytes with overexpression of normal canine NCX [42]. As compared to the original RNC model, the modified model incorporating the Weber *I*_NCX_ produced similar Ca dynamics (S8 Fig). The modified model was validated by comparing the simulated action potential duration at 90% repolarization (APD_90_) under a quasi-steady state after pacing for 100 beats at 1 Hz to experimental data. The computed APD_90_ from the basal model was 185.8 ms, which was in concordance with previous experimental data [18].

### Simulation of HF-induced atrial remodelling

HF-induced atrial remodelling has been identified in various ion channels and Ca^2+^ handling processes. For ion channel remodelling, we focused on HF-induced atrial remodelling associated with a prolonged APD, as observed in studies of dog HF models [18,19,40]. In the atria, it was shown that HF induced (i) a decrease in the transient outward K^+^ current (*I*_to_), slow-delayed rectifier K^+^ current (*I*_Ks_), and *I*_Ca_; (ii) no change in the ultra-rapid-delayed rectifier K^+^ current (*I*_Kur_), rapid-delayed rectifier K^+^ current (*I*_Kr_),_,_ and inward-rectifier K^+^ current (*I*_K1_); (iii) Ca^2+^ handling abnormalities; (iv) fibrosis populations. HF also induced an increase in the SR Ca^2+^ content [18], which was partly attributable to an increased sarcoplasmic reticulum Ca^2+^-ATPase (SERCA) Ca^2+^ reuptake modulated by increased fractional CaMKII phospholamban (PLB) phosphorylation [18]. Therefore, to simulate this increased PLB phosphorylation, the [Ca^2+^]_i_ half-saturation constant for Ca^2+^ uptake into the network SR, *K*_up_, was decreased as in [43]. In addition, Yeh *et al*. [18] showed a decrease in protein levels of SERCA2a and RyR2, a decrease in calsequestrin (Csqn, main SR Ca^2+^-binding protein), and no significant changes of fractional RyR2 phosphorylation.

A summary of HF-induced atrial ion channel and Ca^2+^ handling remodelling in association with prolonged APD at the single cell level is listed in Table 1. HF resulted in an increased APD_90_ and intracellular Ca^2+^ transient (CaT) amplitude matched to experimental observations, thus validating the need to decrease *K*_up_ in simulations [18].

**Table 1:** List of HF-induced atrial ion channels and Ca^2+^ handling remodelling based on data observed in HF dogs and model parameter alterations to simulate HF.

Element	Experimental observation	Model parameters	Change from control
*I*_Ca_	-≈30% [19], -31% (at +10mV) [40]	*G*_Ca_	-30%
*I*_to_	-≈50% [19], -54% (at +40mV) [40]	*G*_to_	-50%
*I*_Ks_	-≈30% [19], -46% (pulse to +50mV) [40]	*G*_Ks_	-45%
*I*_Kur_	not altered [19]	--	not altered
*I*_Kr_	not altered [19]	--	not altered
*I*_K1_	not altered [19,40]	--	not altered
SERCA	Protein levels of SERCA2a -≈35% [18]	*J*_up(max)_	-30%
	Fractional CaMKII phosphorylation of PLB +≈120% [18]	*K*_up_	-78%
RyR2	Protein levels -≈65% [18]	*J*_rel(max)_	-60%
	Fractional RyR2 phosphorylation not significant altered	--	not altered
Csqn	-≈15% [18]	[Csqn]_max_	-15%

Abbreviations: *G*_Ca_, maximal *I*_Ca_ conductance; *G*_to_, maximal *I*_to_ conductance; *G*_Ks_, maximal *I*_Ks_ conductance; NSR, network SR; *J*_up_, Ca^2+^ uptake into the NSR; *K*_up_, [Ca^2+^]_i_ half-saturation constant for *J*_up_; JSR, junctional SR; *J*_rel_, Ca^2+^ release from the JSR; *J*_rel(max)_, maximal Ca^2+^ release rate for *J*_rel_; [Csqn]_max_, total calsequestrin concentration in JSR.

### Parameter sensitivity analysis

Seven parameters were changed from the control condition to simulate the HF-induced atrial remodelling at the single cell model. Multivariable regression with a population of 300 model variants was performed to assess the relative contributions of the different changes of these 7 parameters (Table 1) to the APD_90_, CaT amplitude and SR Ca^2+^ content [44–46]. In addition, each parameter was scaled at a time relative to control or HF conditions, and alternans behavior was assessed to identify the effects of the parameter changes on alternans genesis.

### One-dimensional tissue model

Cell-to-cell electrical coupling in the tissue model can be described via a well-known reaction-diffusion equation [47]. For a 1D model (a cable within cardiac tissue), the mono-domain equation is

$$\frac{\partial V}{\partial t}=-\frac{I_{ion}}{C_{m}}+D\frac{\partial^{2}V}{\partial x^{2}}$$ (1)

where *V* is the membrane potential, *I_ion_* is the total transmembrane ionic current, *C_m_* is the cell capacitance, and *D* is the diffusion coefficient along the 1D fibre.

The 1D simulation was performed on a 50.1 mm strand that consisted of 300 cells with a spatial resolution of 0.167 mm, which was previously used in the canine atria tissue model [48,49]. Stimuli were applied to the left of four cells to evoke AP to propagate along the strand for control and the case when the diffusion coefficient was decreased to 0.028 mm^2^/ms. In the control simulation, the diffusion coefficient *D* was set to 0.167 mm^2^/ms to achieve a conduction velocity (CV) of approximately 0.60 m/s along the cable, which was in line with the CV previously used for tissue simulations [50–52]. The CV was calculated using the method in [53]. It has previously been reported that fibrosis may reduce the cell-to-cell coupling [39]. For HF simulations, the diffusion coefficient *D* was reduced from 0.167 mm^2^/ms to 0.084 mm^2^/ms and 0.028 mm^2^/ms in order to mimic the reduced CVs seen in HF (0.6, 0.4, and 0.2 m/s respectively).

### Simulation protocols

At the single cell level, the dynamic pacing and standard S1-S2 protocols [53,54] were used to analyse the genesis of atrial alternans such as APD_90_ alternans and the intracellular CaT alternans (including the amplitude and decay time), as previously described in detail [55]. The rate dependent curves of APD_90_, CaT amplitude, and CaT decay time against various pacing cycle lengths (PCLs) were obtained by applying a dynamic pacing protocol. The APD_90_ restitution curves against various preceding DIs were obtained by applying the standard S1-S2 protocol.

At the 1D tissue level, the pacing protocol used by Narayan *et al*. [24] was used to induce the alternans. First, steady-state values of the single cell model under control and HF conditions at a PCL of 750 ms were used to initialize all cells in the 1D cable. Then, the 1D cable model was paced at 750 ms for 20 beats. This was followed by pacing for 50 beats at various PCLs.

### Numerical implementation

All time-dependent variables were solved using the Forward Euler method with a time step of 0.02 ms. At the 1D tissue level, a space step was 0.167 mm and a no-flux boundary condition was implemented. Simulations were computed using an Intel Xeon E5-2637 v3 3.50GHz CPU rather than with GPU parallel computing due to increased performance when computing small amounts of cells.

## Results

### Effect of HF-induced electrical remodelling at the single cell level

**Fig 1:**
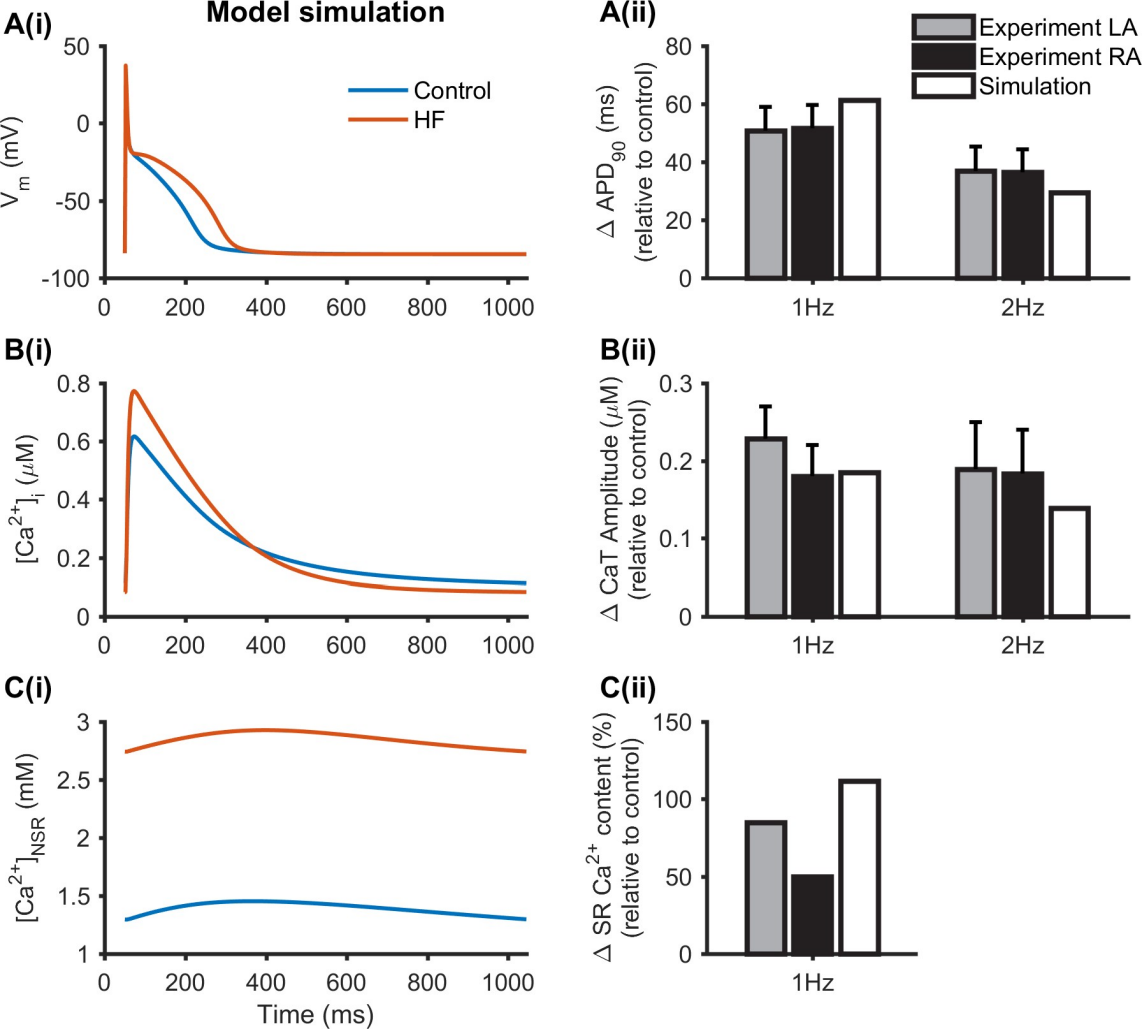
Effect of HF-induced electrical remodelling on the APD_90_, CaT amplitude and SR Ca^2+^ content. (Ai), (Bi) and (Ci) Recordings of APs, intracellular Ca^2+^ concentration ([Ca^2+^]_i_) and Ca^2+^ concentration in NSR ([Ca^2+^]_NSR_) under control and HF conditions at 1Hz. (Aii) and (Bii) The increased APD_90_ (ΔAPD_90_) and increased CaT amplitude (ΔCaT amplitude) under HF conditions relative to control from experimental data [18] (grey and black) and simulations (while) at 1 Hz and 2Hz. (Cii) The increased SR Ca^2+^ content (ΔSR Ca^2+^ content) in HF relative to the control from experimental data [18] (grey and black) and simulations (while) at 1 Hz.

The effects of HF-induced atrial ion channel and Ca^2+^ handling remodelling on the atrial cell APD_90_, CaT amplitude and SR Ca^2+^ content are shown in Fig 1, which were compared to those obtained in the control condition. These simulation results agreed with experimental data from canine heart failure animal model on observations of prolonged APD (Fig 1A) and increased CaT amplitude (Fig 1B) and SR Ca^2+^ content (Fig 1C) [18]. In the simulation, an APD_90_ increase of 61.4 ms was seen in HF cells relative to the control at 1 Hz which was similar to the experimental range of 50.8±8.2 ms in left atria (LA) and 51.6±8.0 ms in right atria (RA) observed in [18], and an APD_90_ increase of 29.6 ms relative to the control at 2 Hz which was within the experimental range of 36.9±8.4 ms in LA and 36.5±7.9 ms in RA observed in [18] (Fig 1A(ii)). HF resulted in an increased CaT amplitude of 185.4 nM and 138.8 nM relative to the control at 1 Hz and 2 Hz (Fig 1B(ii)). This was in agreement with experimental results at 1 Hz (RA: 180.2±40.2 nM) and 2 Hz (LA: 189.2±60.8 nM, RA: 183.6±56.6 nM) observed in [18]. HF produced an increased NSR Ca^2+^ content of 111.6% at 1 Hz, which was near the experimental results at 1 Hz (LA: 85%) observed in [18] (Fig 1C(ii)).

**Fig 2:**
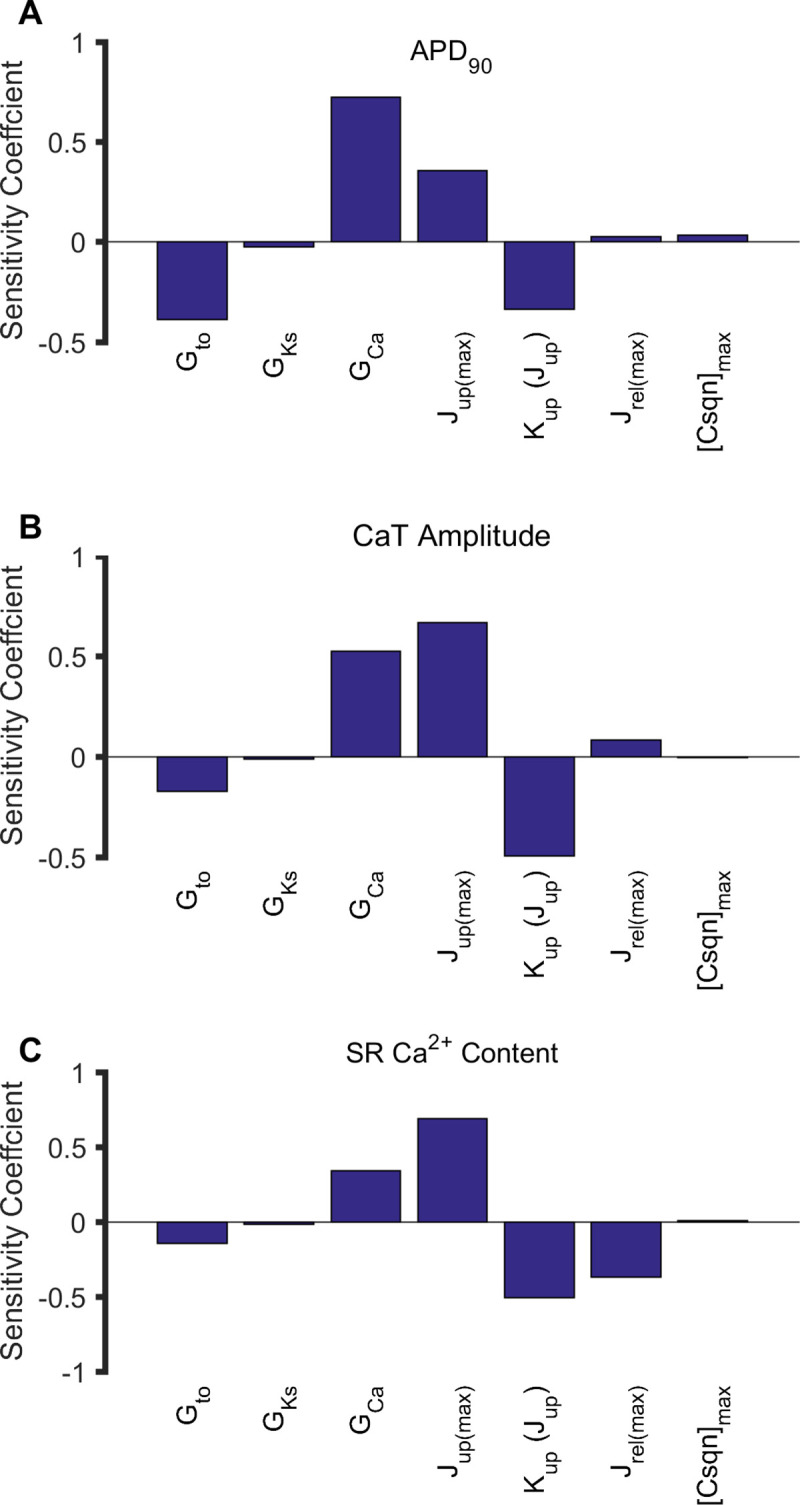
Relative contributions of 7 parameters to the APD_90_, CaT amplitude and SR Ca^2+^ content using parameter sensitivity analysis at 1 Hz. (A) APD_90_. (B) CaT amplitude. (C) SR Ca^2+^ content.

To identify the relative contributions of the 7 identified parameters (Table 1) to the APD_90_, CaT amplitude and SR Ca^2+^ content, parameter sensitivity analysis was performed using multivariable regression based on control model at 1 Hz. The results are shown in Fig 2. Sensitivity coefficients represented the quantitative contribution of each parameter. Increasing *G*_Ca_ and *J*_up(max)_, and decreasing *G*_to_ and *K*_up_ had significant contributions to APD_90_ (Fig 2A), CaT amplitude (Fig 2B) and SR Ca^2+^ content (Fig 2C), of which *G*_Ca_ had the largest contribution to APD_90_, and *J*_up(max)_ had the largest contribution to CaT amplitude and SR Ca^2+^ content. Decreasing *J*_rel(max)_ had a stronger contribution to SR Ca^2+^ content, and increasing *J*_rel(max)_ had only a limited effect on APD_90_ and CaT amplitude. There were minimal effects of *G*_Ks_ and [Csqn]_max_ on APD_90_, CaT amplitude and SR Ca^2+^content. Furthermore, we varied each parameter by different degrees in the HF model one at a time to identify which parameters were the main determinants of HF-induced increases in APD_90_, CaT amplitude and SR Ca^2+^ content (S1 Table). It was shown that *K*_up_ remodelling played an important role in modulating electrical action potentials and Ca^2+^ handling characteristics in HF, as absence of which produced a decrease in APD_90_ and CaT amplitude, and only a small increase in SR Ca^2+^ content relative to control. As compared to the condition when all HF-remodelled factors were considered, absence of *J*_up(max)_ or *G*_Ca_ remodelling produced a larger increase in APD_90_, CaT amplitude and SR Ca^2+^ content; absence of *J*_rel(max)_ remodelling produced a smaller increase in SR Ca^2+^ content, and absence of *G*_to_ remodelling produced a smaller increase in APD_90_ relative to control. These results suggest that HF-induced changes on APD_90_, CaT amplitude and SR Ca^2+^ content are a consequence of all remodelling factors, though the individual role of each is different.

### APD and CaT alternans in HF

**Fig 3:**
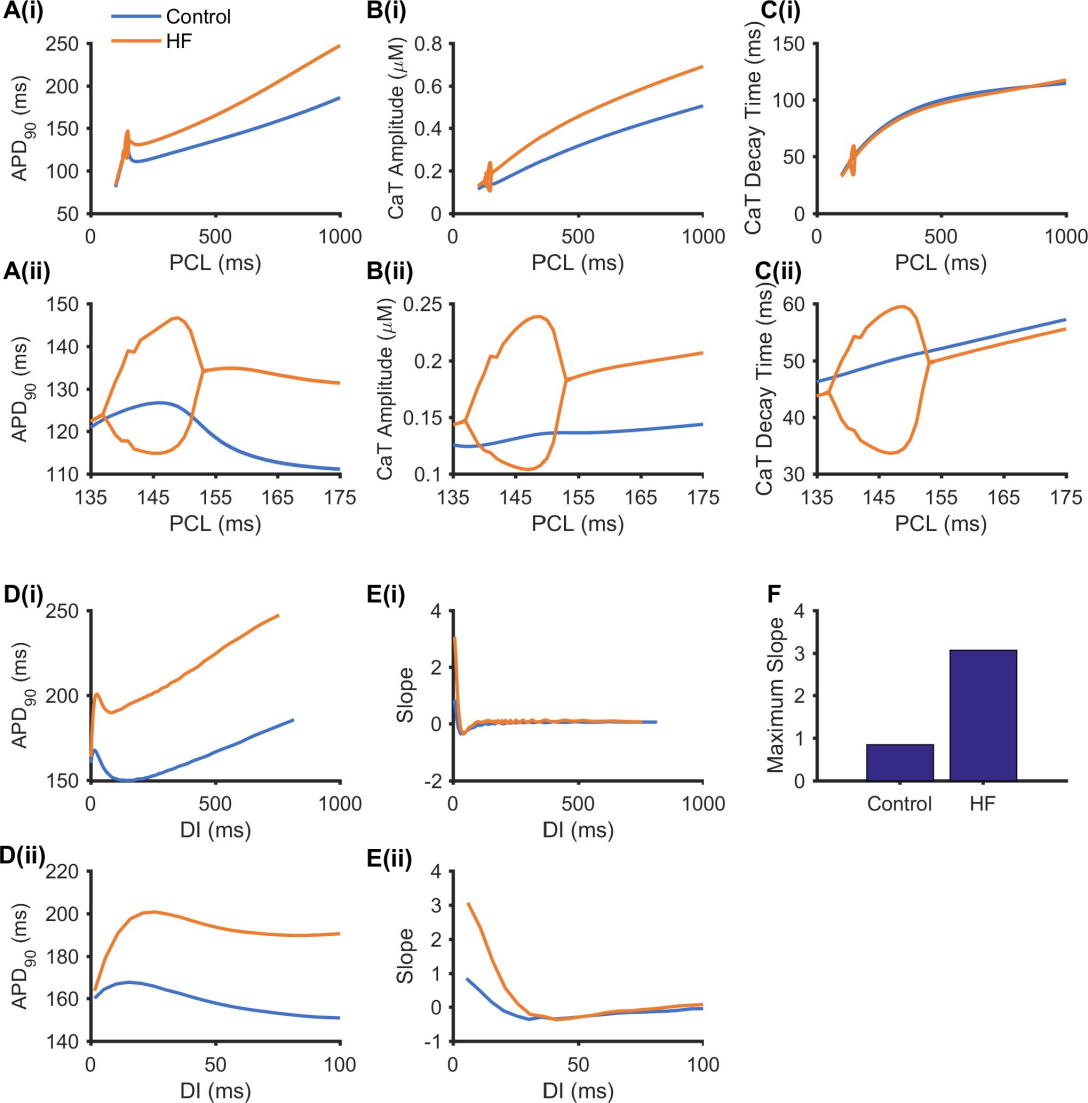
Effect of HF-induced electrical remodelling on rate dependent curves and the restitution curves. (A) APD_90_ rate dependent curves, (B) CaT amplitude rate dependent curves, and (C) CaT decay time rate dependent curves from two consecutive beats in a quasi-steady state under control (shown in blue) and HF (shown in orange) conditions. (D) APD_90_ restitution curves at a quasi-steady state under control (blue) and HF (orange) conditions. (E) Slopes and (F) maximum slope of the restitution curves in (D). (ii) Enlarged view of (i) to more clearly reveal the PCL range with alternans.

Further simulations were conducted to investigate whether HF-induced atrial ion channel remodelling promotes the susceptibility to alternans. Results are shown in Fig 3 for the rate dependent curves of APD_90_ (Fig 3A), CaT amplitude (Fig 3B) and CaT decay time (Fig 3C) obtained from two consecutive beats using the dynamic pacing protocol. Alternans in APD, and CaT amplitude and decay time were observed under HF conditions, but not under the control condition. In HF, the three alternans were characterized as Eye-type alternans [56], indicating an on-setting PCL and a terminating PCL. In the three rate dependent curves, the computed on-setting PCL bifurcation point for alternans genesis was 153 ms under the HF condition (seen in orange in Fig 3A–3C). Under the control condition, the rate dependent curve of the CaT decay time was monotonic, by which the CaT decay time decreased as the PCL decreased (shown in blue in Fig 3C); however, the APD_90_ and CaT amplitude rate dependent curves were biphasic–they first decreased as the PCL decreased from 1000 ms to 180 ms, but then rose at a PCL during 147 ms–180 ms, and decreased again until the PCL reached 100 ms (shown in blue in Fig 3A and 3B). The increased APD_90_ upon decreasing the PCL from 180 ms was due to the supernormal premature action potential [57]. When the PCL was shorter than 180 ms, the stimulus was applied during the relative refractory period, resulting in a small peak action potential and small fast Na^+^ current (*I*_Na_) and *I*_Ca_ that led to small *I*_to_ and *I*_Kur_ values which contributed to slowing repolarization to increase the APD_90_.

It has been suggested that the slope of the APD restitution curve exceeding 1 may provide an indicator for the genesis of APD alternans [30–32], though this theory is limited due to short-term cardiac memory and calcium cycling dynamics [33]. In simulations, the standard S1-S2 protocol was also implemented to compute APD_90_ restitution curves from the dog atrial cell model under control and HF conditions, as well as their maximal slope. Results are shown in Fig 3D–3F. It was shown that the APD_90_ restitution curves under both control and HF conditions were non-monotonic (Fig 3D), as shown in APD_90_ dependent curves under the control condition (Fig 3A). The maximum APD_90_ restitution slope under the control condition was 0.84 (Fig 3E and 3F) which was less than 1; and the maximum slope of APD_90_ restitution in HF was 3.068 (Fig 3E and 3F), which exceeded 1.

**Fig 4:**
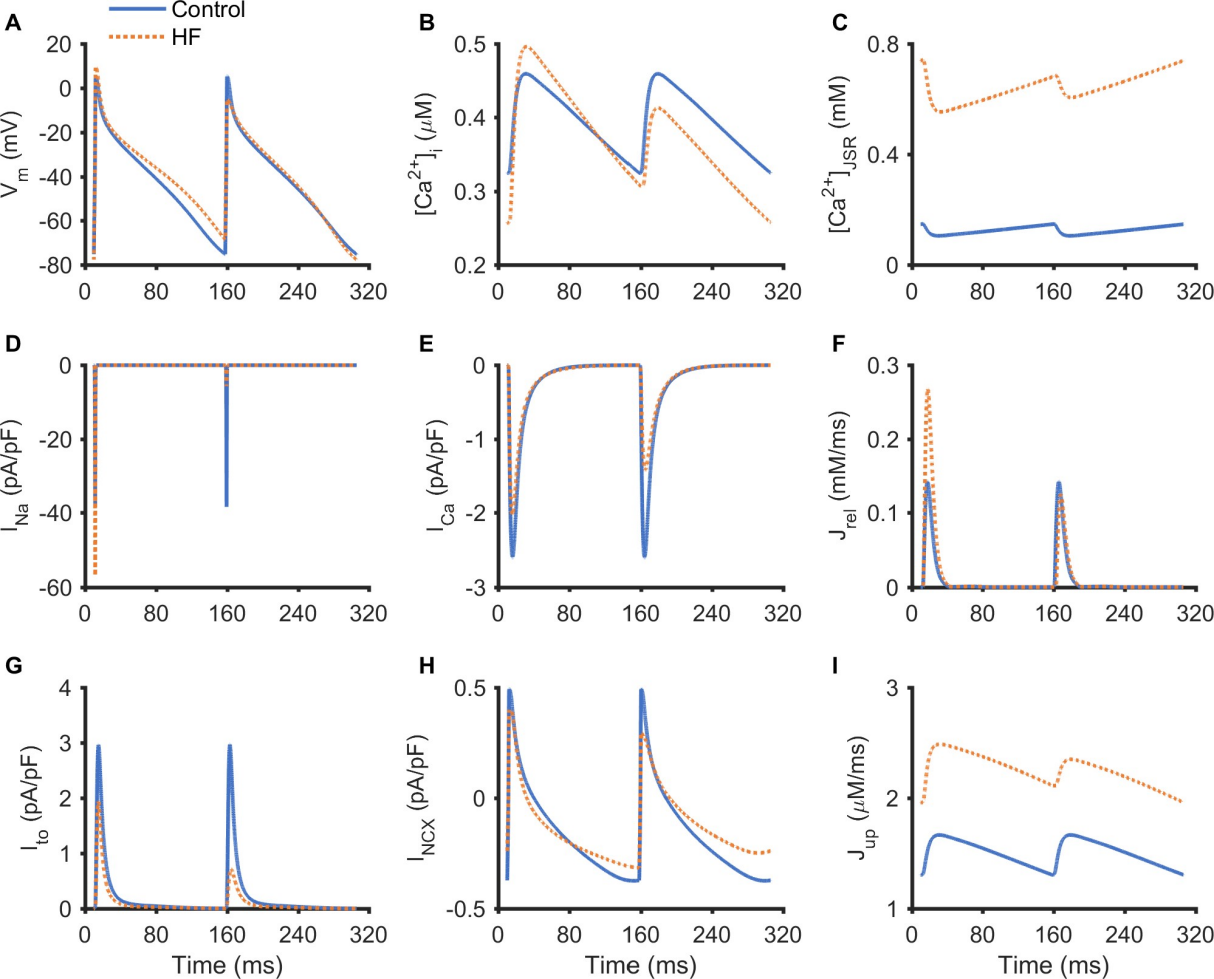
Traces of AP, Ca^2+^ transient, JSR Ca^2+^ concentration and ion channel currents during long-short alternans at a PCL of 148 ms. (A) AP. (B) [Ca^2+^]_i_. (C) JSR Ca^2+^ concentration ([Ca^2+^]_JSR_). (D) *I*_Na_. (E) *I*_Ca_. (F) *J*_rel_. (G) *I*_to_. (H) *I*_NCX_. (I) *J*_up_.

The alternans of AP, Ca^2+^ transient, JSR Ca^2+^ content and ion channel currents are shown in Fig 4 at a PCL of 148 ms. Fig 4A–4C demonstrated alternating APs, Ca^2+^ transients and JSR Ca^2+^ content from two consecutive beats in the HF simulation whereas no alternans was observed in the control. It was shown that the AP alternans was in phase with the Ca^2+^ transient alternans and JSR Ca^2+^ content alternans, manifested by a larger AP amplitude with a longer APD being accompanied by larger CaT amplitude and JSR Ca^2+^ content; and a smaller AP amplitude with a shorter APD accompanied by smaller CaT amplitude and JSR Ca^2+^ content.

**Fig 5:**
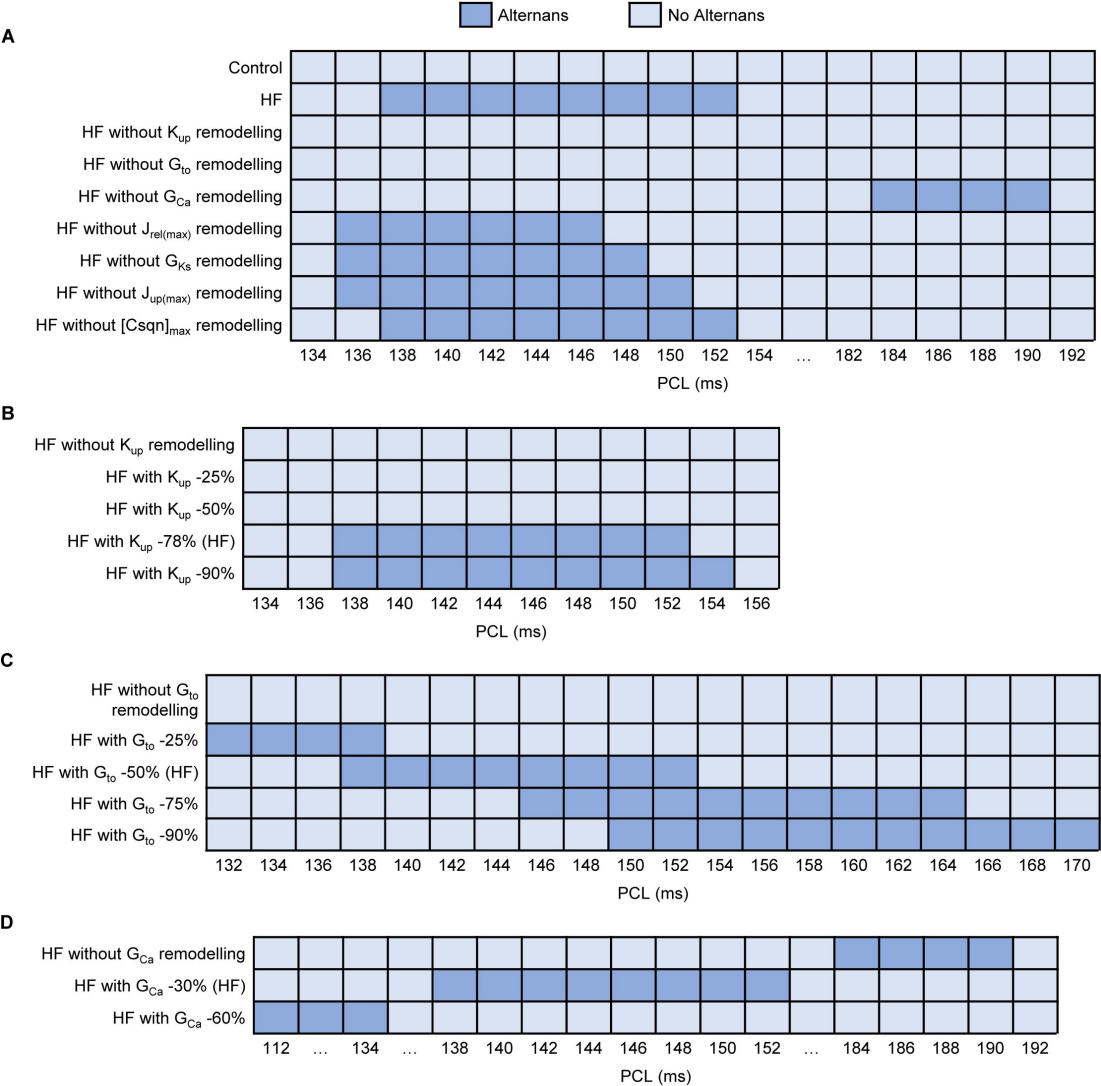
Sensitivity analysis of alternans to HF-induced remodelling in HF model. (A) Each parameter remodelling was removed from the HF model one at a time compared with control and HF models. (B) *K*_up_ was reduced from 0% to 90% relative to the control in the HF model. (C) *G*_to_ was reduced from 0% to 90% relative to the control in the HF model. (D) *G*_Ca_ was reduced from 0% to 60% relative to the control in the HF model.

Further sensitivity analysis of HF-induced remodelling was performed to identify the main determinants for alternans genesis in both control and HF models. Firstly, each HF-induced parameter change was incorporated into the control model one at a time (S2 Fig). In each case, no alternans was observed, suggesting that mechanism for HF-induced alternans was a multi-parameter action. Secondly, each HF-remodelled parameter was removed from the HF model one at a time. Fig 5A demonstrated that HF without *K*_up_ remodelling or *G*_to_ remodelling did not produce alternans, and HF without *G*_Ca_ remodelling produced alternans at longer PCLs. HF without the other 4 remodelled parameters had little effect on alternans (S3 Fig). These results suggested that decreased *K*_up_ and *G*_to_ were the main determinants underlying the occurrence of alternans, and decreased *G*_Ca_ reduced the susceptibility to alternans. Next, each parameter was scaled by different degrees relative to control to investigate how they influenced the occurrence of alternans (*K*_up_, *G*_to_ and *G*_Ca_ in Fig 5B–5D, *J*_rel(max)_, *G*_Ks_, *J*_up(max)_ and [Csqn]_max_ in S3A–S3D Fig). For *K*_up_, alternans occurred in HF until it was decreased by 78%, which may be related to increased SR Ca^2+^ content. As the degree of decreased *G*_to_ gradually increased, alternans occurred at longer PCLs, which may be caused by an increased APD. On the contrary, of the more we reduced *G*_Ca_, the shorter PCLs were when alternans was observed, which may result from decreased APD and CaT amplitude. See Discussion for more details.

### Discordant alternans in the 1D simulation

Conduction of the AP and its corresponding Ca^2+^ transient alternans were also investigated at the 1D tissue level. In simulations, both HF-induced ion channel remodelling and changes in intercellular coupling due to fibrosis (mimicked by a reduced diffusion coefficient, *D*) were considered. Pacing-induced spatial alternans were studied in the 1D simulation by increasing the pacing rate.

**Fig 6:**
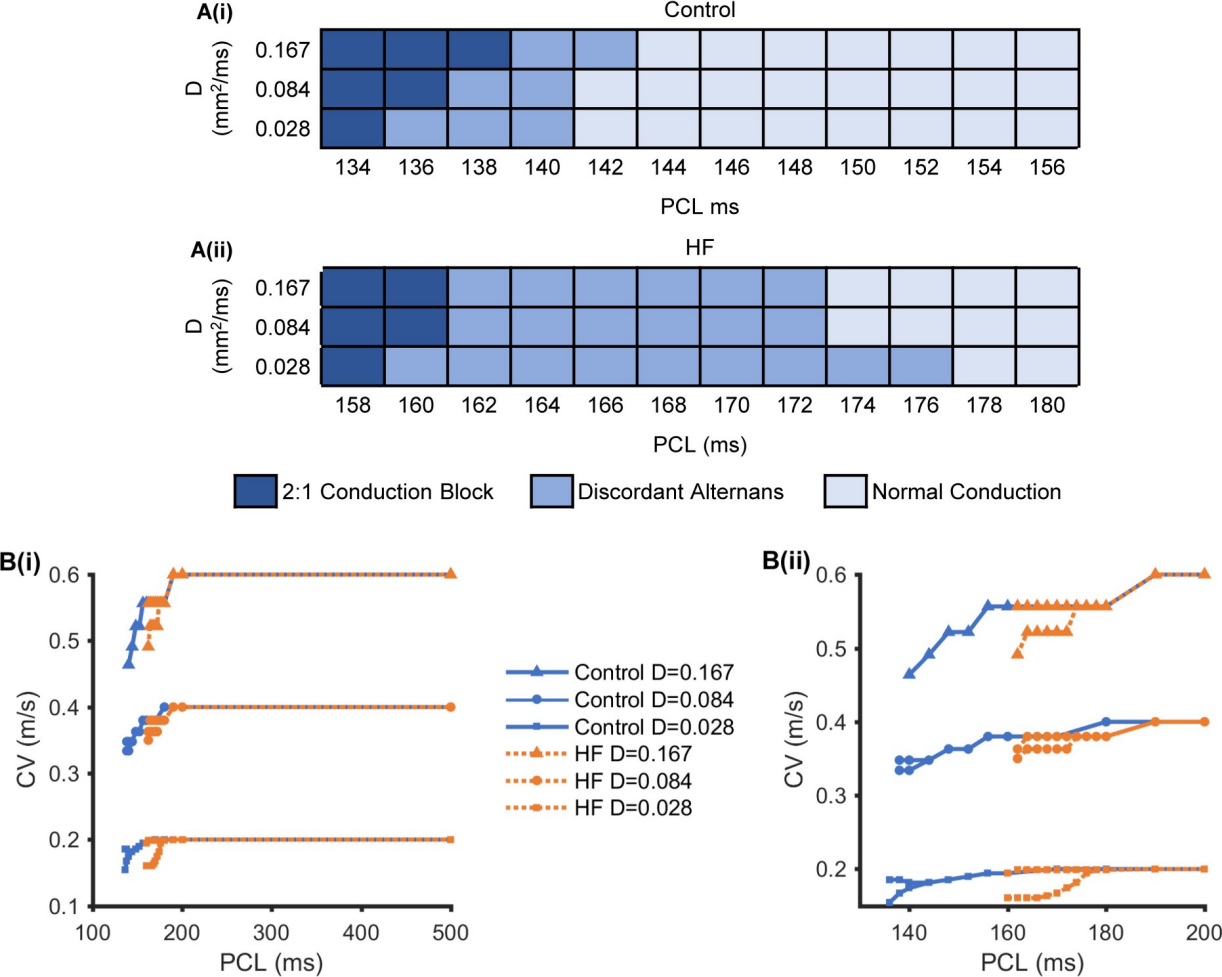
1D simulation results under control and HF conditions. (A) Normal conduction, discordant alternans, and 2:1 conduction block in the *D*-PCL parameter space under control (i) and HF (ii) conditions. (B) The rate dependent curves of CV under control (i) and HF (ii) conditions, as measured by implementing three-member cell-to-cell coupling (*D* = 0.167 mm^2^/ms, 0.084 mm^2^/ms, and 0.028 mm^2^/ms). (Bii) is an enlarged view of (Bi) that more clearly reveals the 130 ms to 200 ms PCL range.

To illustrate the effects of PCLs and cell-to-cell coupling on the genesis of discordant alternans, various AP propagation patterns were investigated in a *D*-PCL parameter space under control and HF conditions, as shown in Fig 6A. There were three AP propagation patterns: normal conduction, discordant alternans, and 2:1 conduction block in a quasi-steady state. The 2:1 conduction block occurred when every other AP was not conducted along the cable. Fig 6B shows rate dependent curves of CV at the 1D tissue level by implementing three cell-to-cell couplings (*D* = 0.028 mm^2^/ms, 0.084 mm^2^/ms, and 0.167 mm^2^/ms) under control and HF conditions. The curves were flat at long PCLs and produced CVs of 0.60 m/s with *D* = 0.167 mm^2^/ms, 0.4 m/s with *D* = 0.084 mm^2^/ms, and 0.2 m/s with *D* = 0.028 mm^2^/ms. They decreased at short PCLs, producing reduced CVs and alternating large and small CVs under both HF and control conditions. The alternating CVs in Fig 6B corresponded to discordant alternans with the same PCLs as in Fig 6A. On the one hand, the PCLs in which discordant alternans occurred were longer under HF conditions than the control condition due to HF-induced APD prolongation. On the other hand, the genesis of discordant alternans was PCL-dependent. When the PCLs were decreased to within a particular range with alternating large and small CVs, discordant alternans occurred under both HF and control conditions. In addition, PCLs in which discordant alternans occurred vary over a wider range when *D* = 0.028 mm^2^/ms than that when *D* = 0.084 mm^2^/ms or 0.167 mm^2^/ms. These suggest that both HF-induced APD prolongation and decreased CVs caused by decreased PCLs and cell-to-cell coupling contribute to the genesis of discordant alternans at the tissue level.

**Fig 7:**
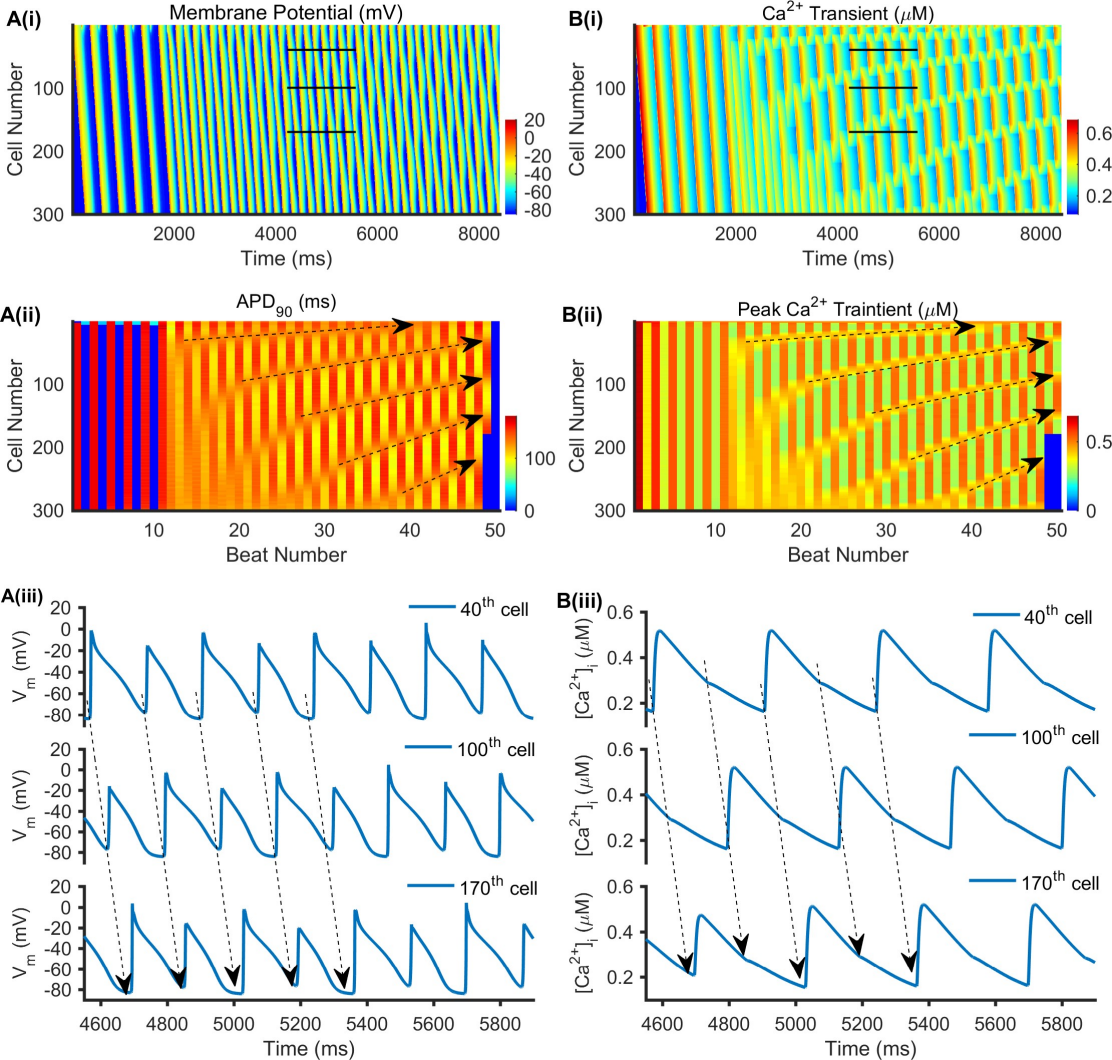
Representative 1D simulation of the discordant alternans with *D* = 0.028 mm^2^/ms at a PCL of 168 ms in HF. (A(i)) Time-space plot of the membrane potential of a homogeneous, 1D cable. (A(ii)) The corresponding APD_90_ of each beat, in which black dashed lines with arrows indicate the APD node position as the beat number increases. (A(iii)) Time course traces of AP for the 40^th^, 100^th^, and 170^th^ cells, which are marked with black lines in (A(i)). (B(i)) Time-space plot of the Ca^2+^ transient in a homogeneous, 1D cable. (B(ii)) The corresponding peak Ca^2+^ transient of each beat, in which black dashed lines with arrows indicate the peak Ca^2+^ transient node position as the beat number increases. (B(iii)) Time course traces of Ca^2+^ transient for the 40^th^, 100^th^, and 170^th^ cells, which are marked with black lines in (B(i)).

Discordant alternans was shown in Fig 7 with *D* = 0.028 mm^2^/ms at a PCL of 168 ms under HF conditions. The AP and Ca^2+^ transient propagation along the cable (vertically from the top 1^st^ cell to the bottom 300^th^ cell) as time increases (horizontally from the left to the right) were colour mapped in Fig 7A(i) and 7B(i), respectively. The APD node, a location between two consecutive beats with same APDs, is an important measurement related to discordant alternans [58]. In Fig 7A(ii), the APD node’s position was indicated by black dashed lines with an arrow, which moved towards the stimulus site (1^st^ cell to 4^th^ cell) as the beat number increased. Along the cable, the alternating areas were out of phase on both sides of the APD node, such as AP traces recorded from the 40^th^, 100^th^, and 170^th^ cells in Fig 7A(iii) (marked by black lines in Fig 7A(i)). APs marked by black dashed lines with arrows exhibited long-short-long-short-long alternans at the 40^th^ cell, short-long-short-long-short alternans at the 100^th^ cell, and long-short-long-short-long alternans at the 170^th^ cell, and these represented discordant alternans. Moreover, the AP and Ca^2+^ transient alternans were in phase (Fig 7A(i) and 7B(i)), with the longer APD_90_ accompanied by a bigger peak Ca^2+^ transient, and the shorter APD_90_ accompanied by a smaller peak Ca^2+^ transient (Fig 7A(ii) and 7B(ii)). This was consistent with observations at the single-cell level (Fig 4A and 4B). In Fig 7B(ii), the position of the peak Ca^2+^ transient node (with little variation and almost the same between two consecutive beats) marked by black dashed lines with an arrow was consistent with the APD nodes in Fig 7A(ii), and also moved towards the stimulus site. As with the AP alternans shown in Fig 7A(iii), the Ca^2+^ transient alternans (marked by black dashed lines with arrows in Fig 7B(iii)) exhibited discordant alternans at the 40^th^, 100^th^, and 170^th^ cells. The larger AP activated the Ca^2+^ transient, but the smaller AP did not as the AP peak potential was insufficient to activate *I*_Ca_ and further activate the Ca^2+^ transient.

Further analyses of the effects of cell-to-cell coupling on the pattern of discordant alternans are shown in Fig 7 with *D* = 0.028 mm^2^/ms and S6 Fig with *D* = 0.167 mm^2^/ms at a PCL = 168 ms under HF conditions. When *D* = 0.167 mm^2^/ms, there was one APD node positioned in a relatively stable manner near the stimulus site (marked with a black arrow in S6A(ii) Fig). However, the number of APD nodes increased when D was reduced to 0.028 mm^2^/ms, and the nodes moved towards the stimulus site with time (Fig 7B(ii)), producing a profound functional spatial heterogeneity. This suggests that cell-to-cell coupling plays an important role in determining the APD node position and number at the 1D tissue level.

**Fig 8:**
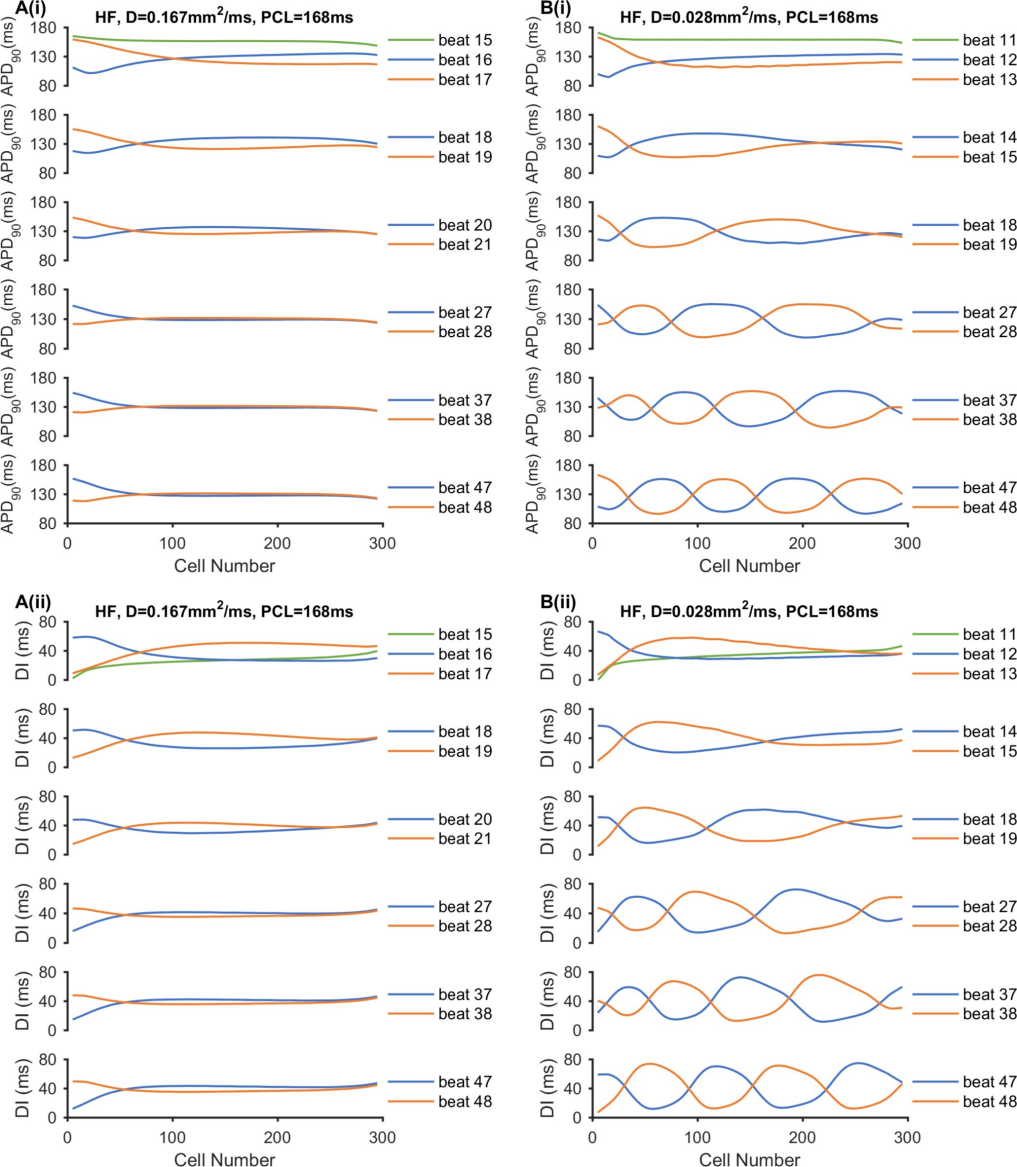
Effect of cell-to-cell coupling on the node position and number with PCL = 168 ms under the HF condition. (A) Results with *D* = 0.167 mm^2^/ms. (B) Results with *D* = 0.028 mm^2^/ms. (i) APD_90_ and (ii) DI spatial distribution for consecutive beats.

Moreover, the pattern of discordant alternans in Fig 7 and S6 Fig can be explained by the evolution of spatial heterogeneity, which are illustrated via the spatial distributions of APD_90_ and DI in Fig 8 and the spatial distribution of the CV in S7 Fig in HF conditions. When D = 0.028 mm^2^/ms, the discordant alternans had two characteristics of nodes moving towards the stimulus site with more nodes forming with continued pacing. This could be interpreted as a larger APD and DI spatial dispersion caused by a slower CV. Reduced PCL and cell-to-cell coupling contributed to decreased CV. For the first two consecutive stimuli (beats 15 and 16 when *D* = 0.167 mm^2^/ms and beats 11 and 12 when *D* = 0.028 mm^2^/ms), the DI node occurred with no APD or CV node. This DI node then produced APD and CV nodes during the second and third stimuli. As the beat number increased, the spatial dispersions of DI and APD_90_ were enhanced for two consecutive beats. A shorter distance was required for the DI and APD_90_ distribution curves to intersect and produce DI and APD nodes. Thus, the nodes gradually moved towards the stimulus site with continued pacing. When *D* = 0.167 mm^2^/ms with a CV of ≈ 0.53 m/s at the CV nodes (S7A Fig), the APD_90_ and DI nodes finally docked at a nearby stimulus site (Fig 8A). When *D* = 0.028 mm^2^/ms with a CV of 0.185 m/s–0.195 m/s at the CV nodes (S7B Fig), the APD and DI dispersion were larger, causing the nodes to move faster (Fig 8B). Additionally, because of small drive force for AP propagation under conditions of weak cell-to-cell coupling, the stability of alternating tissue near the stimulus site could be destroyed, producing nodes disappearing at the stimulus site. Therefore, steeper DI and APD curve slopes promoted faster node movement when the beat number increased, resulting in more node formation.

## Discussion

In this study, the effects of HF-induced atrial remodelling with prolonged APD and decreased cell-to-cell coupling on atrial alternans were investigated *in silico* using the updated canine atrial cell model developed by Ramirez *et al*. [41] at the single cell and 1D tissue level. The simulation results at the single cell level illustrate the effects of HF-induced atrial electrical remodelling (decreased *I*_Ca_, *I*_to_, *I*_Ks_, SRECA2a and RyR2 protein expression, total Csqn, and increased PLB phosphorylation) on the AP and Ca^2+^ transient alternans. Further simulation results at the 1D tissue level demonstrate the effects of HF-induced atrial electrical and structural remodelling (reduced cell-to-cell coupling caused by atrial fibrosis) on spatial alternans. Our major findings follow: (i) The increased SR Ca^2+^ content caused by increased Ca^2+^ uptake by SERCA and decreased RyR2 protein expression contributes to the increased Ca^2+^ transient amplitude, leading to an increase in inward *I*_NCX_. This combined with the decreased *I*_to_ and *I*_Ks_ results in APD prolongation even though with reduced *I*_Ca_ in HF simulations. (ii) The AP and Ca^2+^ transient alternans can be induced in HF simulations, but could not in control simulations, suggesting that HF-induced atrial electrical remodelling can increase the atrial susceptibility to alternans. HF-induced increased PLB phosphorylation and decreased *I*_to_ are the main determinants underlying the occurrence of alternans through [Ca^2+^]_i_→AP coupling and AP→[Ca^2+^]_i_ coupling, respectively. Failure of the increased SR Ca^2+^ content and prolonged AP recovery at the end of diastole underlies the genesis of AP and Ca^2+^ transient alternans in HF. (iii) Cell-to-cell coupling plays an important role in discordant alternans patterns. Reduced cell-to-cell coupling caused by HF-induced atrial fibrosis results in a decreased CV and can enhance spatial dispersion of AP propagation to produce discordant alternans. Together with HF-induced APD prolongation, the enhanced spatial dispersion of AP propagation increases susceptibility to spatial discordant alternans in atrial tissue.

### Model development and relative contribution of parameters in HF simulations

In this study, the effects of HF-induced atrial electrical remodelling were incorporated into the canine atrial cell model. Experimental studies have reported reduced atrial *I*_Ca_ densities in sheep [11], rats [59], dogs [10, 19,40], and humans [17,60,61] with HF. In previous experimental studies, the observations of HF-induced atrial K^+^ current remodelling were not consistent, such as whether *I*_to_ was increased or reduced [10,12,19,40] and whether *I*_Kur_ and *I*_K1_ were unchanged or reduced [10,12,19,40]. In contrast to the inconsistency in *I*_to_, *I*_Kur_ and *I*_K1_ remodelling, *I*_Ks_ has generally been found to decrease in HF [10,19,40] and *I*_Kr_ has been reported as unchanged [19]. The changes of *I*_NCX_ in HF atria were also inconsistent. Li *et al*. [19] and Cha *et al*. [40,62] demonstrated increased transient inward *I*_NCX_ in HF dogs. However, Yeh *et al*. [18] reported no significant changes in NCX1 in control and HF dogs. Given that the increased Ca^2+^ transient peak observed in HF atria can increase the transient inward *I*_NCX_, *I*_NCX_ is likely to be unchanged in HF.

Atrial Ca^2+^ handling abnormalities were also reported in HF, with an increased atrial SR Ca^2+^ content being typically observed in the atria of larger animals and humans [11,17,18,63]. The increased SR Ca^2+^ load could be explained with increased SERCA Ca^2+^ reuptake modulated by increased PLB phosphorylation *via* CaMKII at Thr17 [11,18] or decreased sarcolipin [17,64] in atria with HF, even though the SERCA2a and PLB expressions were unaltered or decreased [11, 17,18,64]. In addition, RyR protein expression and RyR phosphorylation were found to be either unchanged or decreased in large animals and humans with HF [11,17,18,64].

As the computational model used was a canine model, this study focused on reported observations in HF dogs with prolonged APD. The *I*_Ca_, *I*_to_, *I*_Ks_, SERCA2a and RyR2 protein expressions, total Csqn and *K*_up_ were decreased in HF simulations, producing a prolonged APD (S1A Fig) and an increased Ca^2+^ transient amplitude (S1B Fig) and SR Ca^2+^ content (S1D Fig), which are in agreement with experimental results [18]. These are attributable to the integral action of HF-induced remodelling. In spite of a decrease in SERCA2a protein expression, SERCA Ca^2+^ reuptake into the SR was increased (S1H Fig) due to the increased PLB phosphorylation (by decreasing *K*_up_) by CaMKII [18]. This, combined with the decreased RyR2 protein expression and total Csqn, contributed to the increased SR Ca^2+^ content. Even though *I*_Ca_ and RyR2 protein expressions were decreased, the SR Ca^2+^ release (S1F Fig) and CaT amplitude was increased, caused by increased SR Ca^2+^ content. Although it was expected that a decrease in *I*_Ca_ in HF (S1G Fig) might lead to APD shortening, decreases in *I*_to_ and *I*_Ks_ (S1C and S1E Fig) may counteract such an effect. In addition, an increase in CaT amplitude augmented inward *I*_NCX_ and produced APD prolongation, which is consistent with the observations of increased transient inward *I*_NCX_ in previous experimental studies [19,40].

### Mechanism of HF-induced alternans at the single cell level

Previous study in the ventricle has showed that the HF-enhanced genesis of cardiac alternans has been found to be closely related to impairment of Ca^2+^ cycling [65]. In the atria, HF-induced Ca^2+^ handling abnormalities may be related to atrial alternans, including a decrease in *I*_Ca_ and an increase in SR Ca^2+^ content [11,17,18,34–36,63]. In this study, AP, Ca^2+^ transient and JSR Ca^2+^ content alternans were observed in HF simulations, but no alternans were observed in control simulations (Figs 3 and 4), suggesting that HF-induced remodelling enhances susceptibility to cardiac alternans. While numerous clinical and animal experiments and simulations on ventricles have explored the underlying mechanism of alternans [31,34,66–68], there is still controversy whether abnormalities in AP or Ca^2+^ handling is the predominant factor [39]. The results in this study suggest that *K*_up_ and *G*_to_ remodelling are the main determinants underlying the occurrence of atrial alternans. This demonstrates that both abnormalities of AP and Ca^2+^ handling may play important role in alternans.

HF-induced an increase in PLB phosphorylation was simulated by decreasing *K*_up_ ([Ca^2+^]_i_ half-saturation constant for Ca^2+^ uptake into the network SR), and the underlying mechanism of alternans could be supported by [Ca^2+^]_i_→AP coupling in HF. Decreasing *K*_up_ resulted in an increase in SERCA Ca^2+^ reuptake (S4A Fig), leading to increasing the SR Ca^2+^ content (S4B Fig). Increases in the SR Ca^2+^ content produced larger SR Ca^2+^ release (S4C Fig) and Ca^2+^ transients (S4D Fig). When the PCL was decreased to 148 ms, SR Ca^2+^ concentrations could not be restored to the level at the end of diastole that led to a smaller SR Ca^2+^ release and Ca^2+^ transient in the next beat. This caused beat-to-beat alternation in the intracellular Ca^2+^ transient (S4D Fig), and therefore induced *I*_NCX_ alternans (S4E Fig). Therefore, in two consecutive pacing cycles, a larger inward *I*_NCX_ contributed to slower repolarization in one beat while a smaller inward *I*_NCX_ contributed to faster repolarization in the following beat, forming the alternans of AP repolarization (APD alternans) as shown in S4F Fig. Beat-to-beat alternation in APD alternans caused beat-to-beat alternation in the *I*_Na_ (S4G Fig) and *I*_Ca_ (S4H Fig), as the AP could not be restored to basal level at the end of diastole when the APD was longer. Further, the alternans of *I*_Ca_ acted as a positive feedback on the alternans of the Ca^2+^ transient amplitude via Ca^2+^-induced Ca^2+^ release from SR.

Another AP→[Ca^2+^]_i_ coupling can explain the underlying mechanism by which HF-induced decreases in *I*_to_ (simulated by decreasing *G*_to_) increases the susceptibility to alternans in HF. Decreasing *G*_to_ (S5A Fig) resulted in an increase in the APD (S5B Fig). When the PCL was decreased to 148 ms, the AP could not be restored to the level at the end of diastole that led to a smaller AP in the next beat, forming the beat-to-beat alternation in AP and APD. AP alternans caused alternans of the *I*_Na_ (S5C Fig) and *I*_Ca_ (S5D Fig). *I*_Ca_ alternans was then responsible for alternans of both SR Ca^2+^ release (S5E Fig) and Ca^2+^ transients (S5F Fig). Further, the plateau phase of the AP was elevated and prolonged by reducing *G*_to_, which led to increased transmembrane Ca^2+^ influx through *I*_Ca_ (S5D Fig) resulting in SR Ca^2+^ accumulation (S5G Fig), in agreement with experimental research [19]. The increased SR Ca^2+^ content also played a key role in alternans of SR Ca^2+^ concentrations and Ca^2+^ transients as described above. Moreover, the alternans of Ca^2+^ transient caused alternans of *I*_NCX_ (S5H Fig) which in turn affected APD alternans.

### Mechanism of HF-induced discordant alternans at the cable tissue level

Spatially discordant alternans in atria underlies the development of atrial arrhythmia [25,39,69] and is characterized by opposite alternating phases at different areas of tissue. In this study, we demonstrated the effects of the PCL and cell-to-cell coupling on spatial discordant alternans in control and HF conditions. Only discordant alternans were observed in this study. When the PCLs was decreased to a certain range, discordant alternans occurred with different cell-to-cell coupling (*D* = 0.167 mm^2^/ms, 0.084 mm^2^/ms, and 0.028 mm^2^/ms) in both control and HF conditions (Fig 8). Compared with strong cell-to-cell coupling (*D* = 0.167 mm^2^/ms), additional APD nodes were produced under conditions with weak cell-to-cell coupling (*D* = 0.028 mm^2^/ms), and the range of PCLs at which discordant alternans occurred was longer especially in HF conditions (Figs 6 and 7, and S6 Fig).

Patterns of discordant alternans vary with the strength of cell-to-cell coupling. (i) The DI and APD nodes moved towards the stimulus site as pacing continued (Fig 8), which is consistent with observations by Watanabe *et al*. [58]. With strong cell-to-cell coupling, the DI and APD nodes finally docked at a site near the stimulus site (Fig 8A). However, these nodes moved much faster before disappearing at stimulus sites with weak cell-to-cell coupling (Fig 8B). Alternans occurred at the stimulus site and propagated along the cable. With strong cell-to-cell coupling, the driving force for AP propagation was larger and created relatively stable in-phase alternans near the stimulation site (Fig 8A). Even if the DI and APD nodes move towards the stimulation site, they are unable to destroy the stability near the stimulation site. In contrast, if the cell-to-cell coupling is weak and the driving force is small, stability near the stimulation site can be destroyed where DI and APD nodes move to the stimulus site and will disappear (Fig 8B). (ii) The number of DI and APD nodes increased when cell-to-cell coupling was weak (Fig 8B). This can be interpreted as reduced intercellular coupling enhancing the dispersion of AP distribution between adjacent myocytes [39], producing steeper DI and APD distribution curves. This promotes faster node movement as the beat number increases, resulting in more node formation. Furthermore, experimental results from rabbit heats demonstrated that the gap junction modifier rotigaptide could reduce the repolarization heterogeneity and suppress the discordant alternans [70]. This adds evidence of the significant role of cell-to-cell coupling in the genesis of discordant alternans, which contributes to increased susceptibility to arrhythmias.

### Limitations

HF-induced atrial electrical remodelling varies with various stages and complications of cardiac disease [8,9] and various experimental techniques and conditions [6]. Simulation in this study focused on HF with prolonged APD, which was observed in dog atria. Further research should be undertaken to investigate whether HF with unaltered or shortened APD also makes a contribution to atrial arrhythmia. The canine atrial cell model developed by Ramirez *et al*. [41] did not incorporate the CaMKII regulatory pathway. For this reason, *K*_up_ was decreased to simulate the increased Ca^2+^ reuptake by SECRA which is modulated by increased PLB phosphorylation due to CaMKII as in [43]. Though some preliminary studies have been done here (S1 File), but detailed studies warrant to be conducted in the future to update the canine atrial cell model with more detailed calcium handling and CaMKII regulatory pathway based on atrial experimental data.

For the 1D tissue model, we only considered the homogeneous tissue and did not consider heterogeneous tissue. Cell-to-cell coupling disrupted by HF-induced fibrosis was also only included in the 1D tissue simulation. This warrants further studies by incorporating the fibroblast model coupled with atrial myocytes and the arrangement of fibroblasts in atrial tissue. Furthermore, the 1D tissue model should be extended to 2D or 3D tissue model to explore the transition from discordant alternans to re-entry in HF.

Despite these limitations, the canine HF model we developed in the present study can simulate experimental observations in cellular electrical activities in HF dogs, such as the prolonged APD. The developed model may be incorporated into a computational platform in future for further investigations of pro-arrhythmic effects of HF in the atria, particularly with respect to atrial alternans, results of which may provide new insights into the clinical treatment of AF with HF.

## Supporting information

S1 Fig Effect of HF-induced electrical remodelling on AP, Ca^2+^ transient, JSR Ca^2+^ concentration and ion channel currents at 1 Hz and 2 Hz. (A) AP. (B) [Ca^2+^]_i_. (C) *I*_to_. (D) Junctional SR Ca^2+^ concentration ([Ca^2+^]_JSR_). (E) *I*_Ks_. (F) *J*_rel_. (G) *I*_Ca_. (H) *J*_up_. (I) *I*_NCX_.
(TIF)

S2 Fig Sensitivity analysis of alternans to HF-induced remodelling in control model. Each parameter remodelling induced by HF was incorporated into the control model at a time.
(TIF)

S3 Fig Sensitivity analysis of alternans to each parameter remodelling reduced by 0% to 90% relative to control at a time in the HF model. (A) *J*_rel(max)_, (B) *G*_Ks_, (C) *J*_up(max)_, and (D) [Csqn]_max_ were reduced from 0% to 90% relative to the control in the HF model.
(TIF)

S4 Fig Traces of AP, Ca^2+^ transient, JSR Ca^2+^ concentration and ion channel currents during long-short alternans at a PCL of 148 ms in the HF model with *K*_up_ reduced by 0% to 78% relative to control at a time. (A) *J*_up_. (B) [Ca^2+^]_JSR_. (C) *J*_rel_. (D) [Ca^2+^]_i_. (E) *I*_NCX_. (F) AP. (G) *I*_Na_. (H) *I*_Ca_.
(TIF)

S5 Fig Traces of AP, Ca^2+^ transient, JSR Ca^2+^ concentration and ion channel currents during long-short alternans at a PCL of 148 ms in the HF model without *G*_to_ remodelling and with *G*_to_ reduced by 50% relative to control at a time. (A) *I*_to_. (B) AP. (C) *I*_Na_. (D) *I*_Ca_. (E) *J*_rel_. (F) [Ca^2+^]_i_. (G) [Ca^2+^]_JSR_. (H) *I*_NCX_.
(TIF)

S6 Fig Representative 1D simulation of the discordant alternans accompanied by conduction block with *D* = 0.167 mm^2^/ms at a PCL of 168 ms under the HF condition. (A(i)) Time-space plot of the membrane potential of a homogeneous, 1D cable. (A(ii)) The corresponding APD_90_ of each beat, in which black dashed lines with arrows indicate the APD node position as the beat number increases. (B(i)) Time-space plot of the Ca^2+^ transient in a homogeneous, 1D cable. (B(ii)) The corresponding peak Ca^2+^ transient of each beat, in which black dashed lines with arrows indicate the peak Ca^2+^ transient node position as the beat number increases.
(TIF)

S7 Fig CV spatial distribution for consecutive beats at PCL = 168ms under the HF condition. (A) Results with *D* = 0.167 mm^2^/ms. (B) Results with *D* = 0.028 mm^2^/ms.
(TIF)

S8 Fig Comparison between the Weber (dashed line) and RNC (solid line) *I*_NCX_ models on AP, Ca^2+^ transient, JSR Ca^2+^ concentration and ion channel currents at 1 Hz and 2 Hz. (A) AP. (B) [Ca^2+^]_i_. (C) *I*_NCX_. (D) [Ca^2+^]_JSR_. (E) *J*_rel_. (F) *J*_up_.
(TIF)

S1 Table Relative contribution of each parameter by varying different degrees in HF model at a time. The values of APD_90_, CaT amplitude, and SR Ca^2+^ content were computed relative to control, and the bold font demonstrated the HF model.
(PDF)

S1 File Supplementary materials for methods and results. (DOCX)
