# Supplementary figures and images for: Heart failure-induced atrial remodelling promotes electrical and conduction alternans
Source: PLoS Comput Biol. 2020 Jul 13;16(7):e1008048. doi: 10.1371/journal.pcbi.1008048 (PMC7402519; doi:10.1371/journal.pcbi.1008048)

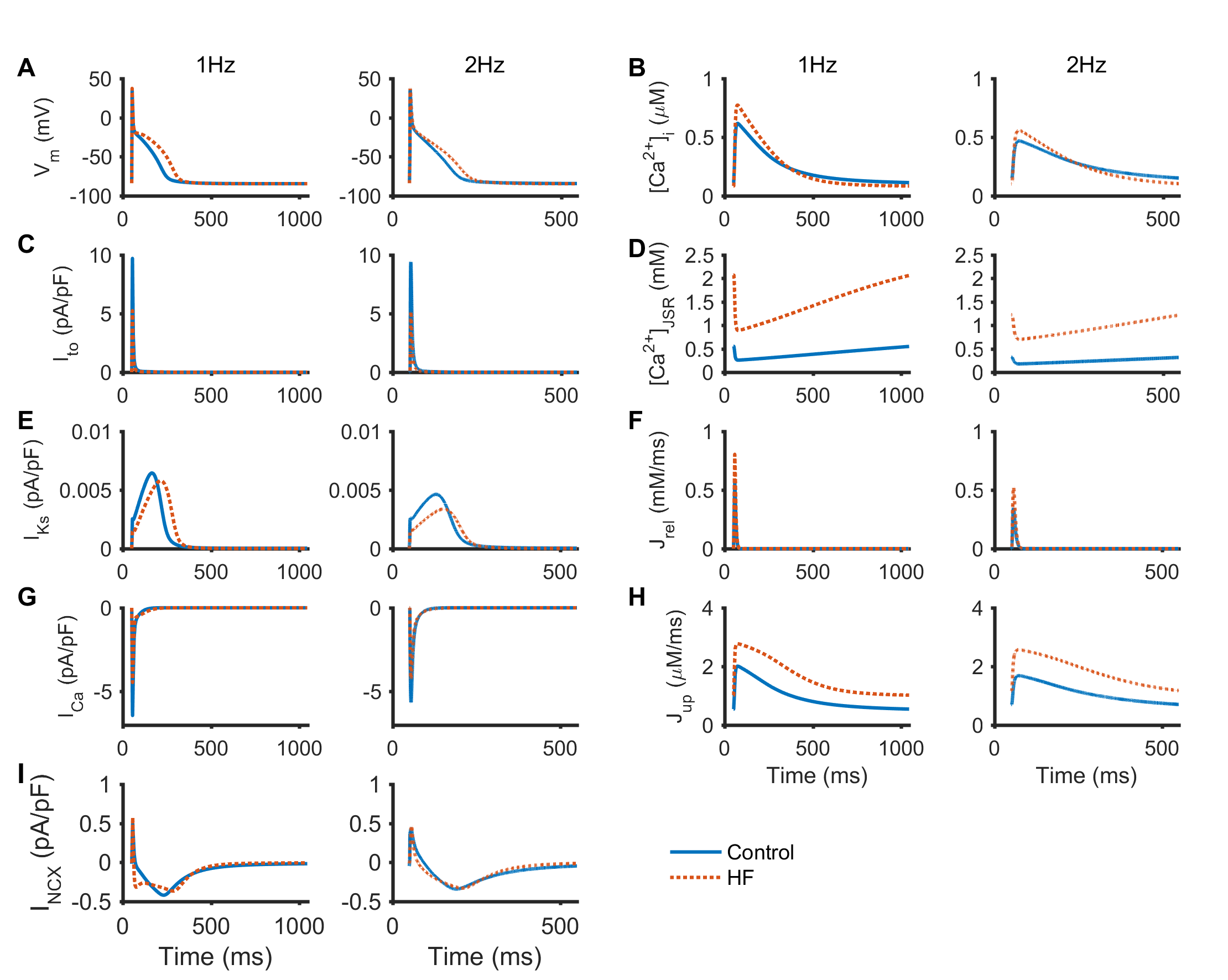

Supplement: S1 Fig — (A) AP. (B) [Ca2+]i. (C) Ito. (D) Junctional SR Ca2+ concentration ([Ca2+]JSR). (E) IKs. (F) Jrel. (G) ICa. (H) Jup. (I) INCX. (TIF) [file pcbi.1008048.s001.tif]

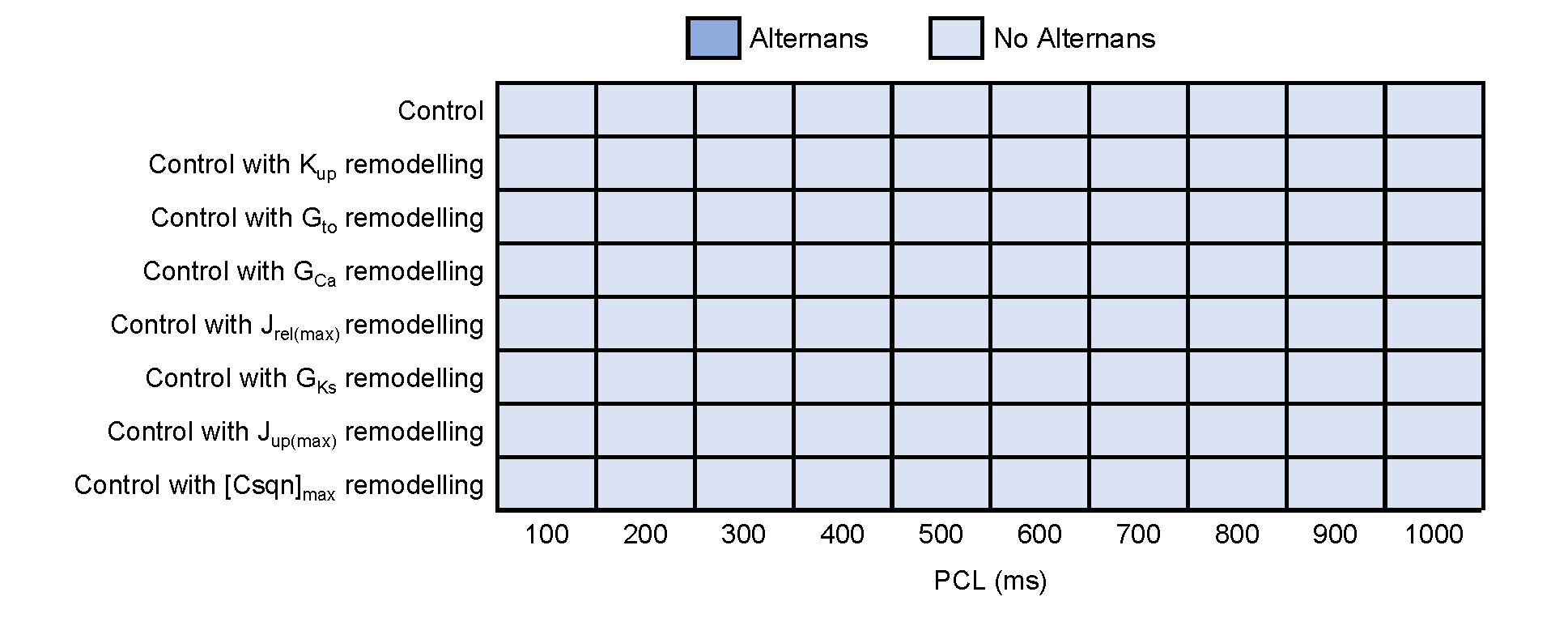

Supplement: S2 Fig — Each parameter remodelling induced by HF was incorporated into the control model at a time. (TIF) [file pcbi.1008048.s002.tif]

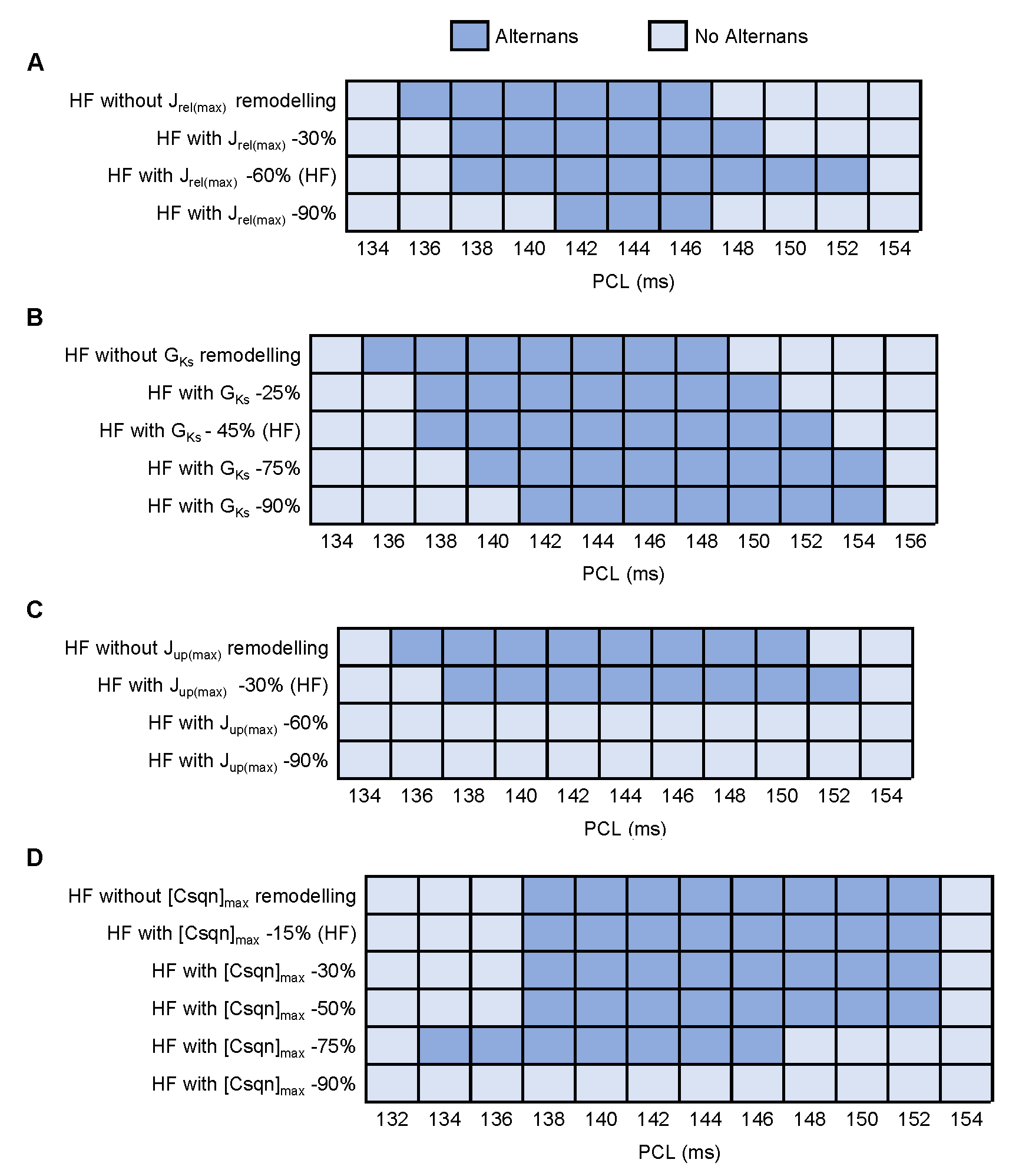

Supplement: S3 Fig — (A) Jrel(max), (B) GKs, (C) Jup(max), and (D) [Csqn]max were reduced from 0% to 90% relative to the control in the HF model. (TIF) [file pcbi.1008048.s003.tif]

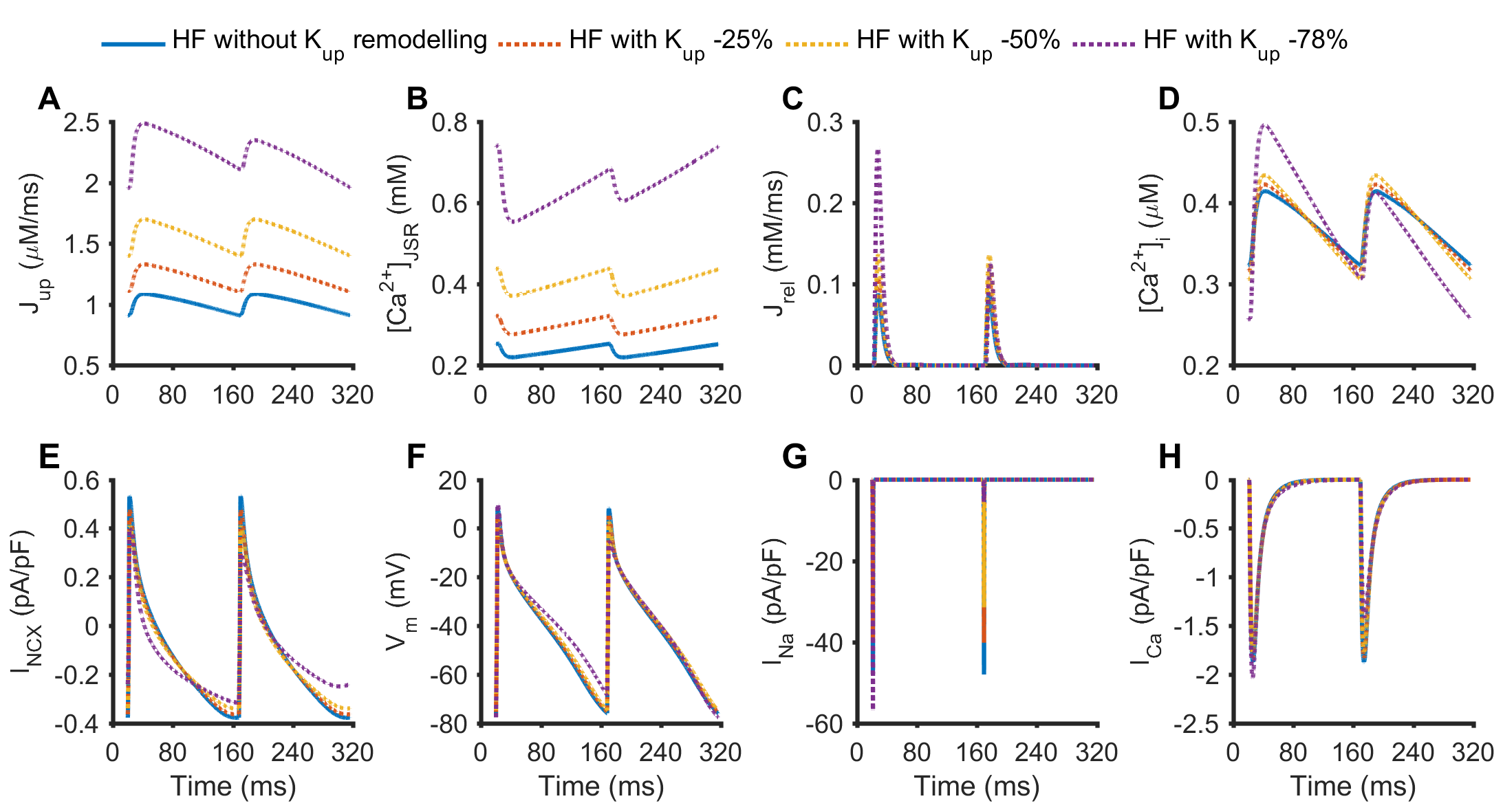

Supplement: S4 Fig — (A) Jup. (B) [Ca2+]JSR. (C) Jrel. (D) [Ca2+]i. (E) INCX. (F) AP. (G) INa. (H) ICa. (TIF) [file pcbi.1008048.s004.tif]

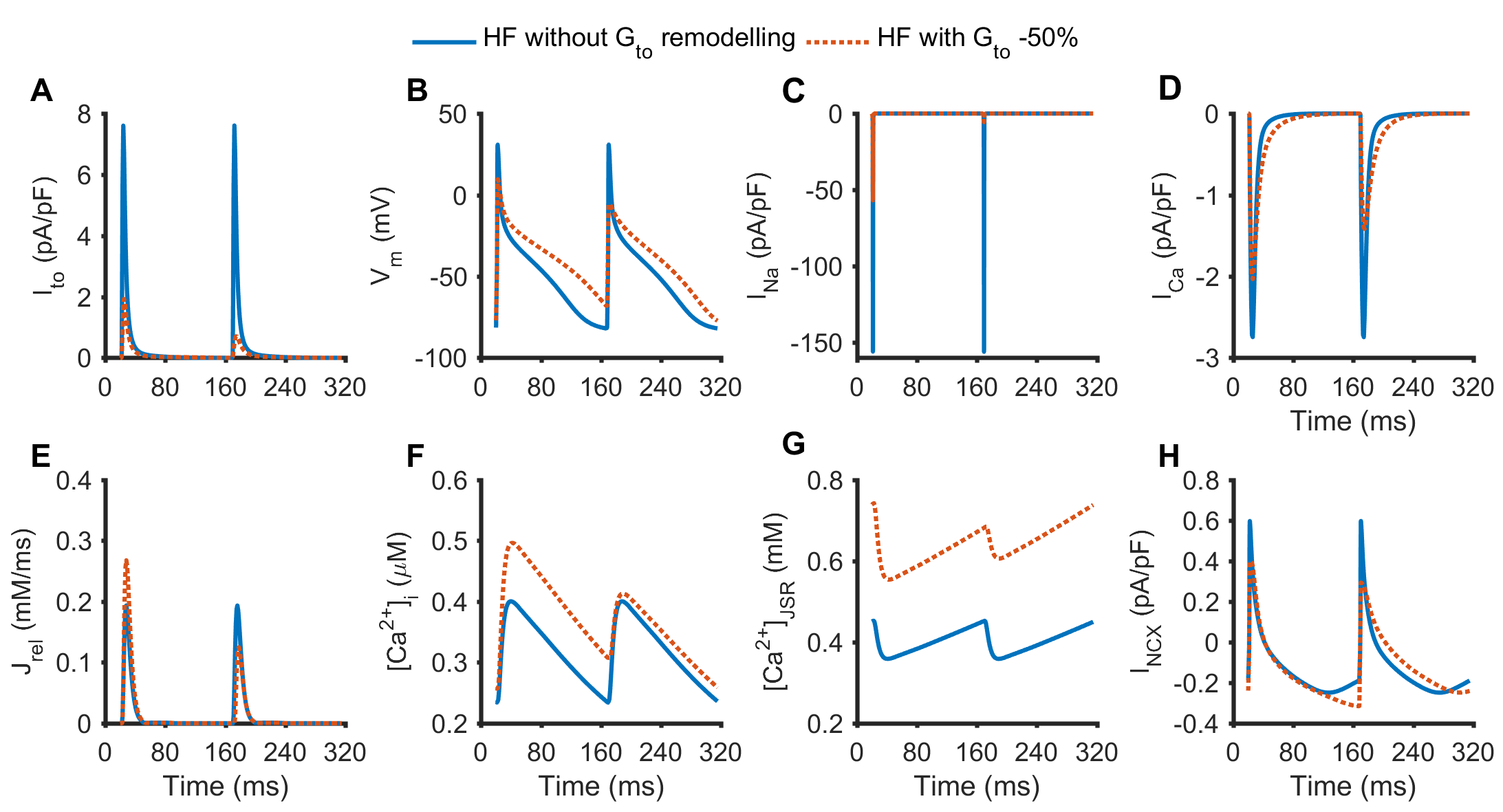

Supplement: S5 Fig — (A) Ito. (B) AP. (C) INa. (D) ICa. (E) Jrel. (F) [Ca2+]i. (G) [Ca2+]JSR. (H) INCX. (TIF) [file pcbi.1008048.s005.tif]

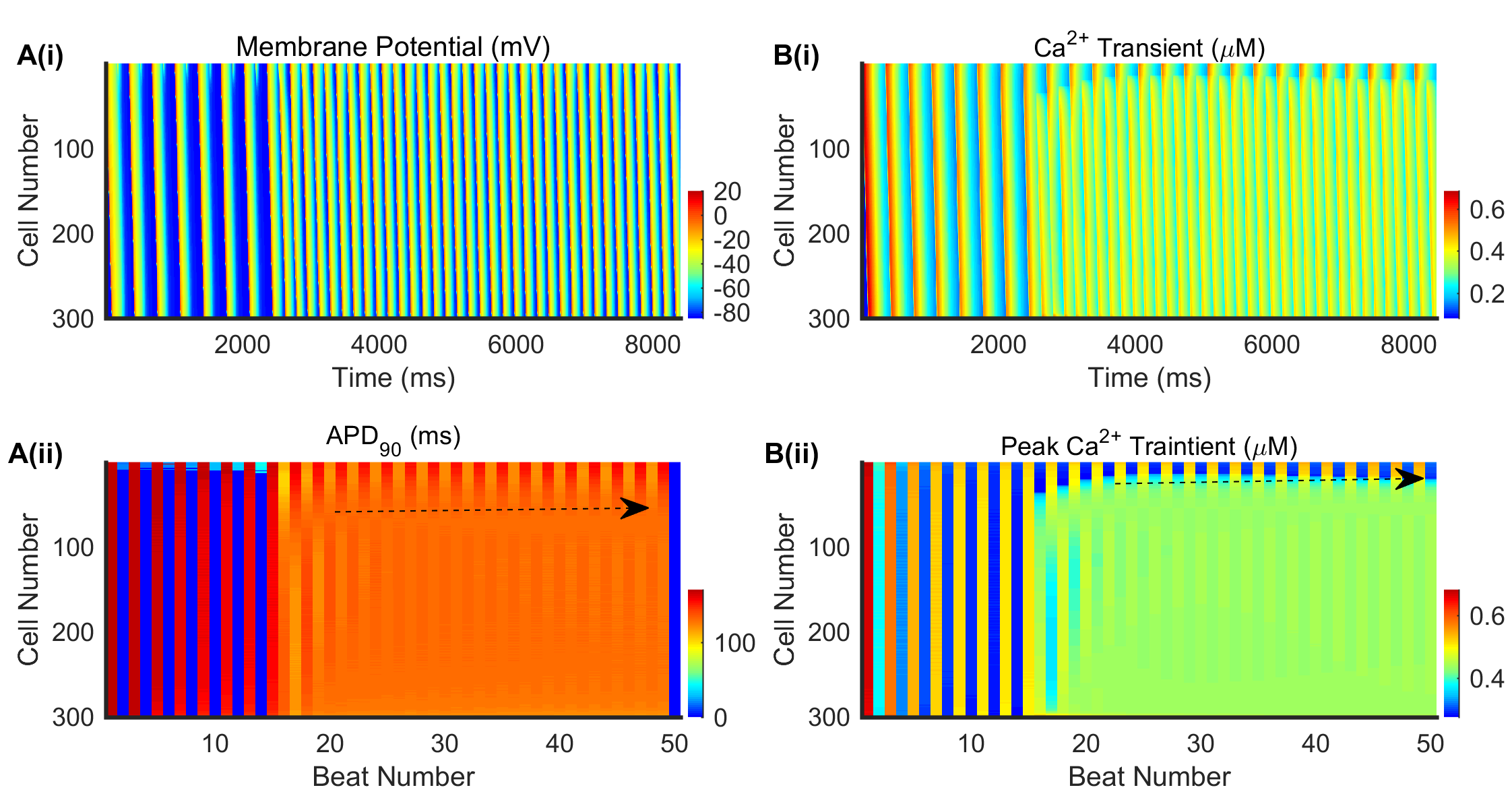

Supplement: S6 Fig — (A(i)) Time-space plot of the membrane potential of a homogeneous, 1D cable. (A(ii)) The corresponding APD90 of each beat, in which black dashed lines with arrows indicate the APD node position as the beat number increases. (B(i)) Time-space plot of the Ca2+ transient in a homogeneous, 1D cable. (B(ii)) The corresponding peak Ca2+ transient of each beat, in which black dashed lines with arrows indicate the peak Ca2+ transient node position as the beat number increases. (TIF) [file pcbi.1008048.s006.tif]

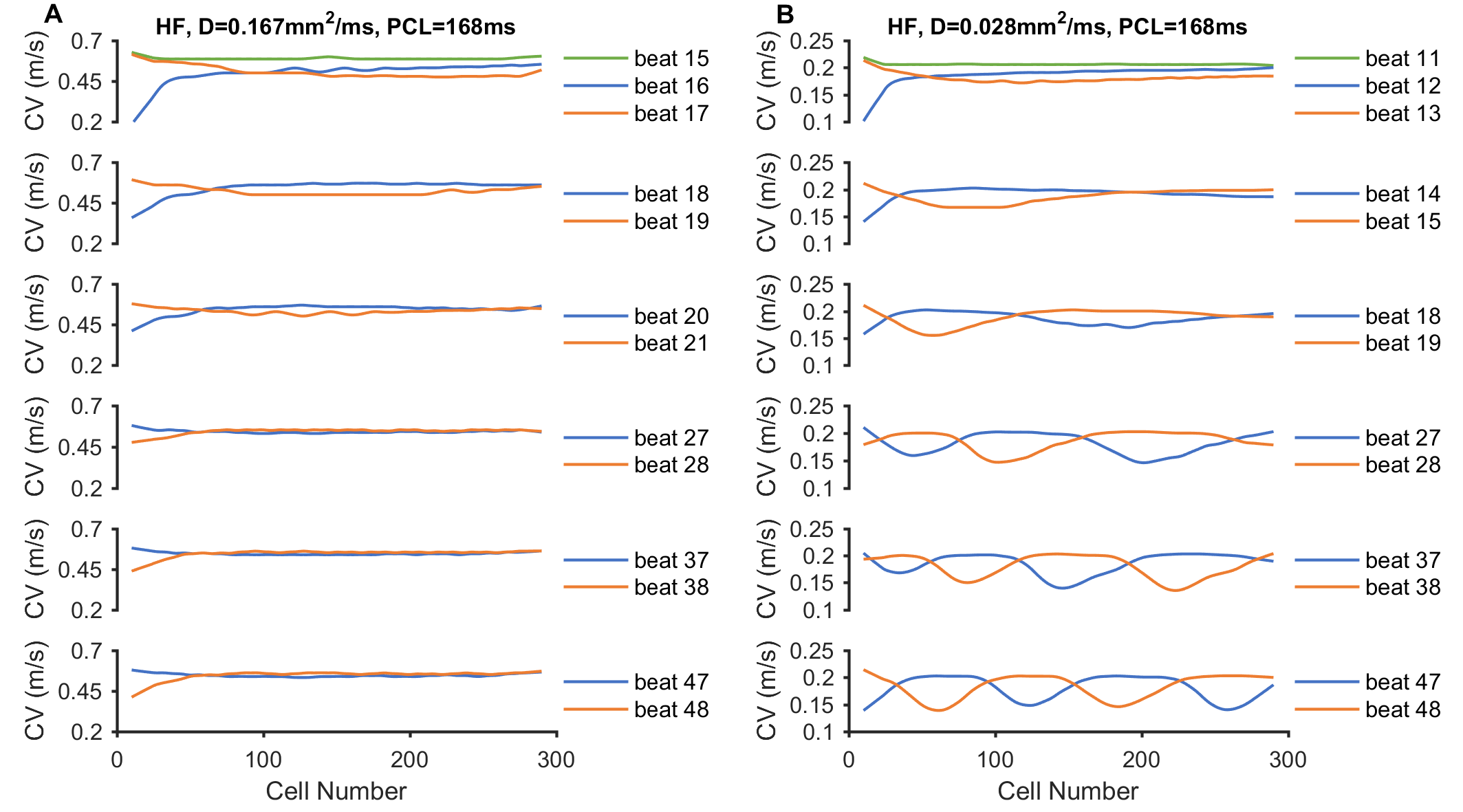

Supplement: S7 Fig — (A) Results with D = 0.167 mm2/ms. (B) Results with D = 0.028 mm2/ms. (TIF) [file pcbi.1008048.s007.tif]

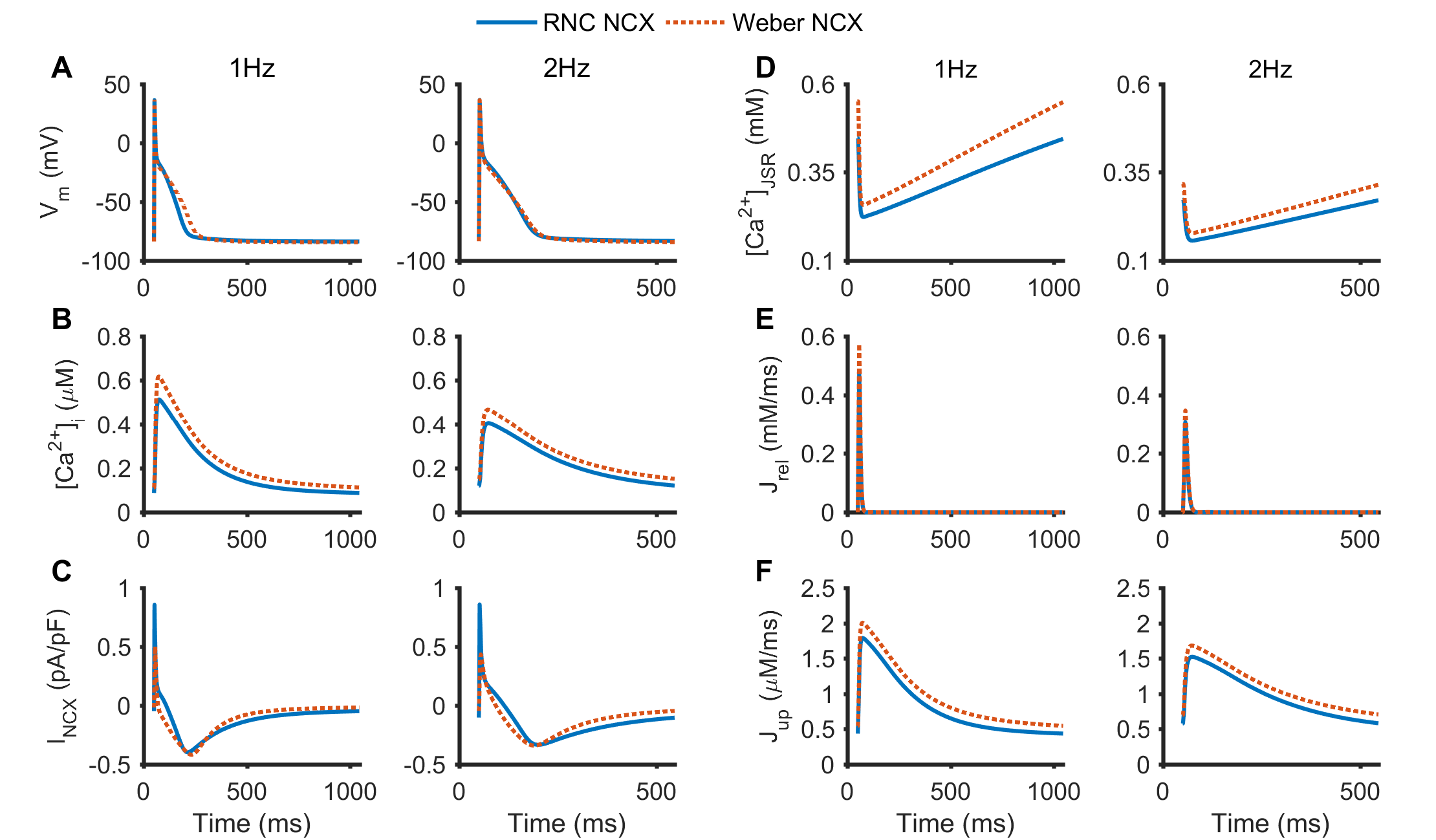

Supplement: S8 Fig — (A) AP. (B) [Ca2+]i. (C) INCX. (D) [Ca2+]JSR. (E) Jrel. (F) Jup. (TIF) [file pcbi.1008048.s008.tif]
