# Supplementary material for: Heart failure-induced atrial remodelling promotes electrical and conduction alternans
Source: PLoS Comput Biol. 2020 Jul 13;16(7):e1008048. doi: 10.1371/journal.pcbi.1008048 (PMC7402519; doi:10.1371/journal.pcbi.1008048)
Supplement: S1 Table — The values of APD90, CaT amplitude, and SR Ca2+ content were computed relative to control, and the bold font demonstrated the HF model. (PDF) [file pcbi.1008048.s009.pdf]

S1 Table. Relative contribution of each parameter by varying different degrees in HF model at a time. The values of APD_{90}, CaT amplitude and SR Ca^{2+} content were computed relative to control, and the bold font demonstrated the HF model.

Parameters	Relative to control	APD_{90}	CaT amplitude	JSR Ca^{2+} content
K_{up}	-90%	+52.7%	+73.0%	+424.3%
	**-78%**	**+ 33.0%**	**+36.7%**	**+275.6%**
	-65%	+13.3%	+10.1%	+178.8%
	-50%	-1.5%	-9.6%	+115.5%
	-25%	-14.7%	-29.4%	+56.3%
	-0%	-21.3%	-41.4%	+ 22.7%
$J_{up(max)}$	-50%	+7.9%	+ 0.8%	+143.4%
	**-30%**	**+ 33.0%**	**+36.7%**	**+275.6%**
	-10%	+44.1%	+72.4%	+431.3%
	-0%	+45.3%	+88.7%	+511.2%
G_{Ca}	-50%	+14.3%	+22.2%	+251.0%
	**-30%**	**+ 33.0%**	**+36.7%**	**+275.6%**
	-10%	+51.7%	+54.1%	+316.3%
	-0%	+59.5%	+62.5%	+338.1%
$J_{rel(max)}$	-80	+17.7%	+ 7.46%	+441.1%
	**-60%**	**+ 33.0%**	**+36.7%**	**+275.6%**
	-40%	+37.8%	+50.0%	+187.8%
	-0%	+40.7%	+61.8%	+101.7%
G_{to}	-70%	+37.9%	+40.4%	+291.0%
	**-50%**	**+ 33.0%**	**+36.7%**	**+275.6%**
	-30%	+ 25.2%	+ 31.9%	+256.4%
	-0%	+8.9%	+24.2%	+227.7%
G_{Ks}	-65%	+33.5845%	+36.8497%	+276.1151%
	**-45%**	**+ 33.0463%**	**+36.6987%**	**+275.5902%**
	-25%	+32.5188%	+36.5502 %	+275.0745%
	-0%	+31.8%	+36.3%	+274.4%
$[Csqn]_{max}$	-30%	+31.7869%	+36.6476%	+274.9764%
	**-15%**	**+ 33.0463%**	**+36.6987%**	**+275.5902%**
	-5%	+ 33.6383%	+36.7197%	+275.8681%
	-0%	+33.8%	+36.7%	+275.9%
