# Supplementary material for: Heart failure-induced atrial remodelling promotes electrical and conduction alternans
Source: PLoS Comput Biol. 2020 Jul 13;16(7):e1008048. doi: 10.1371/journal.pcbi.1008048 (PMC7402519; doi:10.1371/journal.pcbi.1008048)
Supplement: S1 File — (DOCX) [file pcbi.1008048.s010.docx]

Supplementary materials for methods and results

**S1. Incorporating the CaMKII model**

We have tried to incorporated the CaMKII model from Hund et al., 2008 [1] and Christensen et al., 2009 [2] respectively into the updated canine atrial cell model by Ramirez *et al.* [3] (denoted as RNC-Hund 08 model and RNC-Hund 09 model). The targets of CaMKII included *I*_NaL_, *I*_Ca_, PLB and RyR2. HF-induced ion channel remodelling and Ca^2+^ handling abnormalities were described in Table 1 which added the increased CaMKII isoform. Yeh et al., 2008 [5] demonstrated that although the expression the cytosolic CaMKIIδ isoform increased by 123%, the fractional RyR2 phosphorylation was not significant altered in HF. Therefore, in the HF model, increased CaMKII effect of increased CaMKIIδ isoform on RyR2 was removed.

Table 1. List of HF-induced ion channel and Ca^2+^ handling remodelling based on data observed in HF dogs and model parameters alterations to simulate HF.

| **Element** | **Experimental observation** | **Model parameters** | **Change from control** |
| --- | --- | --- | --- |
| *I*_Ca_ | -≈30% [4], -31% (at +10mV) [5] | *G*_Ca_ | -30% |
| *I*_to_ | -≈50% [4], -54% (at +40mV) [5] | *G*_to_ | -50% |
| *I*_Ks_ | -≈30% [4], -46% (pulse to +50mV) [5] | *G*_Ks_ | -45% |
| *I*_Kur_ | not altered [4] | -- | not altered |
| *I*_Kr_ | not altered [4] | -- | not altered |
| *I*_K1_ | not altered [4] | -- | not altered |
| SERCA | Protein levels of SERCA2a -≈35% [6] | *J*_up(max)_ | -30% |
|  | Fractional CaMKII phosphorylation of PLB +≈120% [6] | Δ*K*_PLB,CaMK_ | +120% |
| RyR2 | Protein levels -≈65% [6] | *J*_rel(max)_ | -60% |
|  | Fractional RyR2 phosphorylation not significant altered | -- | not altered |
| Csqn | -≈15% [6] | [Csqn]_max_ | -15% |
| CaMKII | Expression of cytosolic CaMKIIδ isoform +123% [6] | CaMK_0_ in [1]  camlbar in [2] | +123% |

Abbreviations: CaMK_0_, fraction of active CaMKII binding sites at equilibrium; camlbar, free calmodulin concentration; Δ*K*_PLB,CaMK_, CaMKII dependence to [Ca^2+^]_i_ half-saturation constant for *J*_up_, and using (*K*_up_ - Δ*K*_PLB,CaMK_) to simulate the CaMKII effect on PLB.

Firstly, in model simulation, the expression of CaMKIIδ isoform was set to increase by 123%. The results showed shortened action potential (AP), decreased Ca^2+^ transient (CaT) amplitude and SR Ca^2+^ content (Fig 1A). Then, the expression of CaMKIIδ isoform was set to increase by 200%. The results also showed shortened AP, decreased CaT amplitude and JSR Ca^2+^ content (Fig 1B). Fig 1A(iii) and 1B(iii) suggested that only increasing CaMKII expression could not result in increasing CaMKII phosphorylation of PLB by 120% to produce increased SERCA Ca^2+^ uptake in spite of reduced SERCA2a protein expression in HF, which was observed in Yeh et al., 2008 [5].

As the two CaMKII model did not include how to calculate the CaMKII phosphorylation levels at targets, Δ*K*_PLB,CaMK_ was increased by from 100% to 400% to simulate increasing CaMKII phosphorylation of PLB based on the expression of CaMKIIδ isoform increased by 123%. The results in Fig 2 showed that Δ*K*_PLB,CaMK_ increased by 300% to 400% produced increased SERCA Ca^2+^ uptake and JSR Ca^2+^ content, but no increase in APD and CaT amplitude, which did not match the experimental data in Yeh et al., 2008 [5].

In summary, when the CaMKII model from Hund et al., 2008 [1] or Christensen et al., 2009 [2] was incorporated into the updated RNC model, HF-induced ion channel and Ca^2+^ handling remodelling based on experimental data [4-6] could not result in prolonged APD and increases in CaT amplitude and SR Ca^2+^ content. This may be caused by the following reasons.

1. The CaMKII model from Hund et al., 2008 [1] or Christensen et al., 2009 [2] was canine ventricular model and based on experimental data in ventricle. Directly incorporating a CaMKII model may need to adjust based on atrial experimental data, in spite of a few studies of atrial myocytes [7]. And this is beyond the scope of this article.
2. The targets of the two CaMKII model included *I*_NaL_, *I*_Ca_, PLB and RyR2. Other studies suggested that CaMKII may have effect on *I*_K1_ and *I*_to_ [8,9], which were not included in the two CaMKII model.
3. Yeh et al., 2008 [5] showed that the expression the cytosolic CaMKIIδ isoform increased by 123% and fractional CaMKII phosphorylation of PLB increased by 120%, but fractional RyR2 phosphorylation was not significant altered in HF. This indicates that increased expression the CaMKIIδ isoform enhancing CaMKII phosphorylation of all targets is uncertain. And CaMKII phosphorylation of *I*_NaL_ and *I*_Ca_ in HF is unknown.
4. The two CaMKII model did not include how to calculate the CaMKII phosphorylation levels at targets. And it is unclear whether CaMKIIδ isoform increased by 123% produces CaMKII phosphorylation of PLB increased by 120%.

Therefore, we did not incorporate the CaMKII model into the updated RNC model for this study.


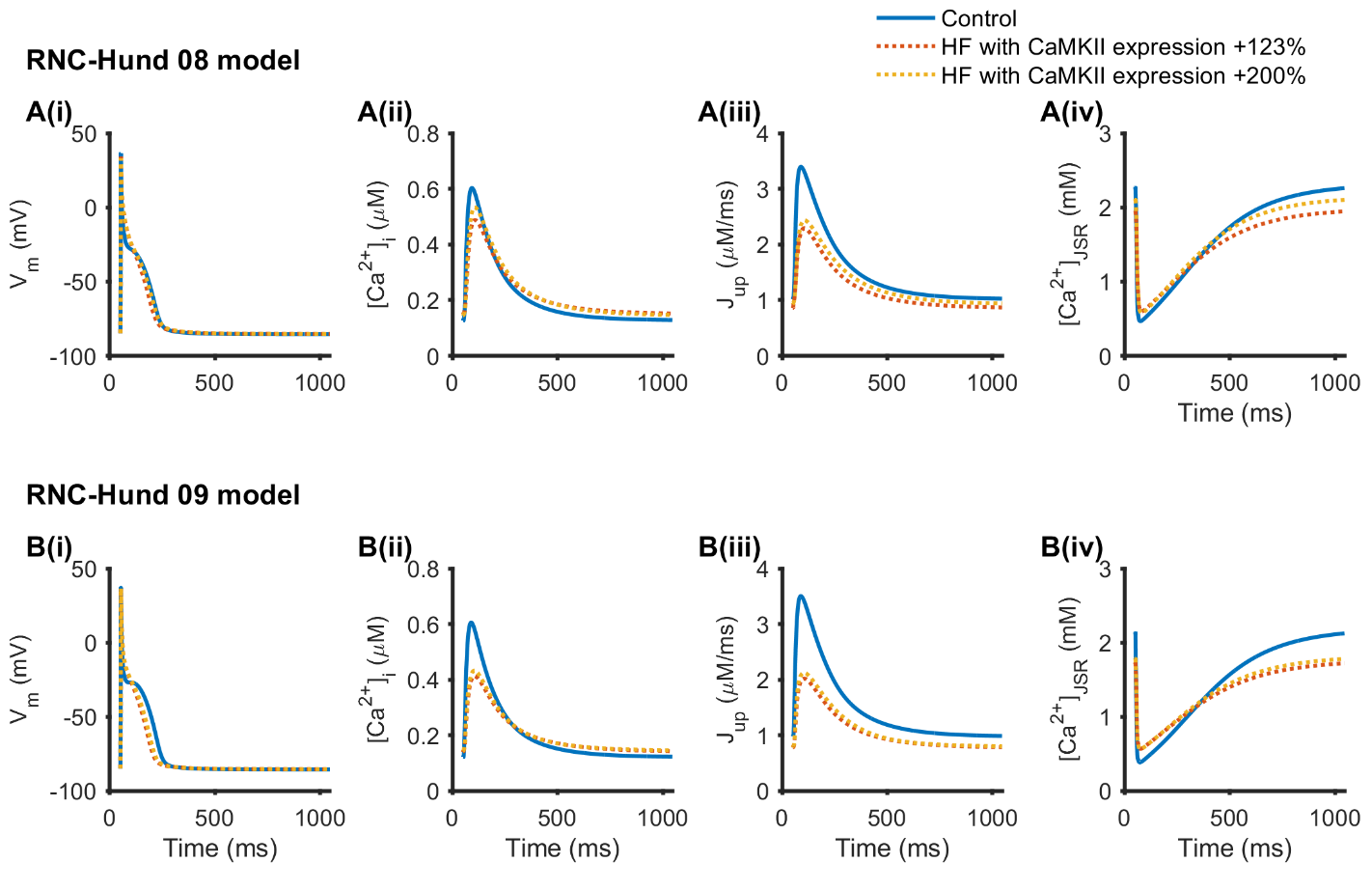


Fig 1. Effect of HF-induced electrical remodelling with the expression of CaMKIIδ isoform increased by 123% and 200% on the AP (i), Ca^2+^ transient (ii), SERCA Ca^2+^ uptake (*J*_up_) (iii) and JSR Ca^2+^ content (iv) in RNC-Hund 08 model (A) and RNC-Hund 09 model (B).


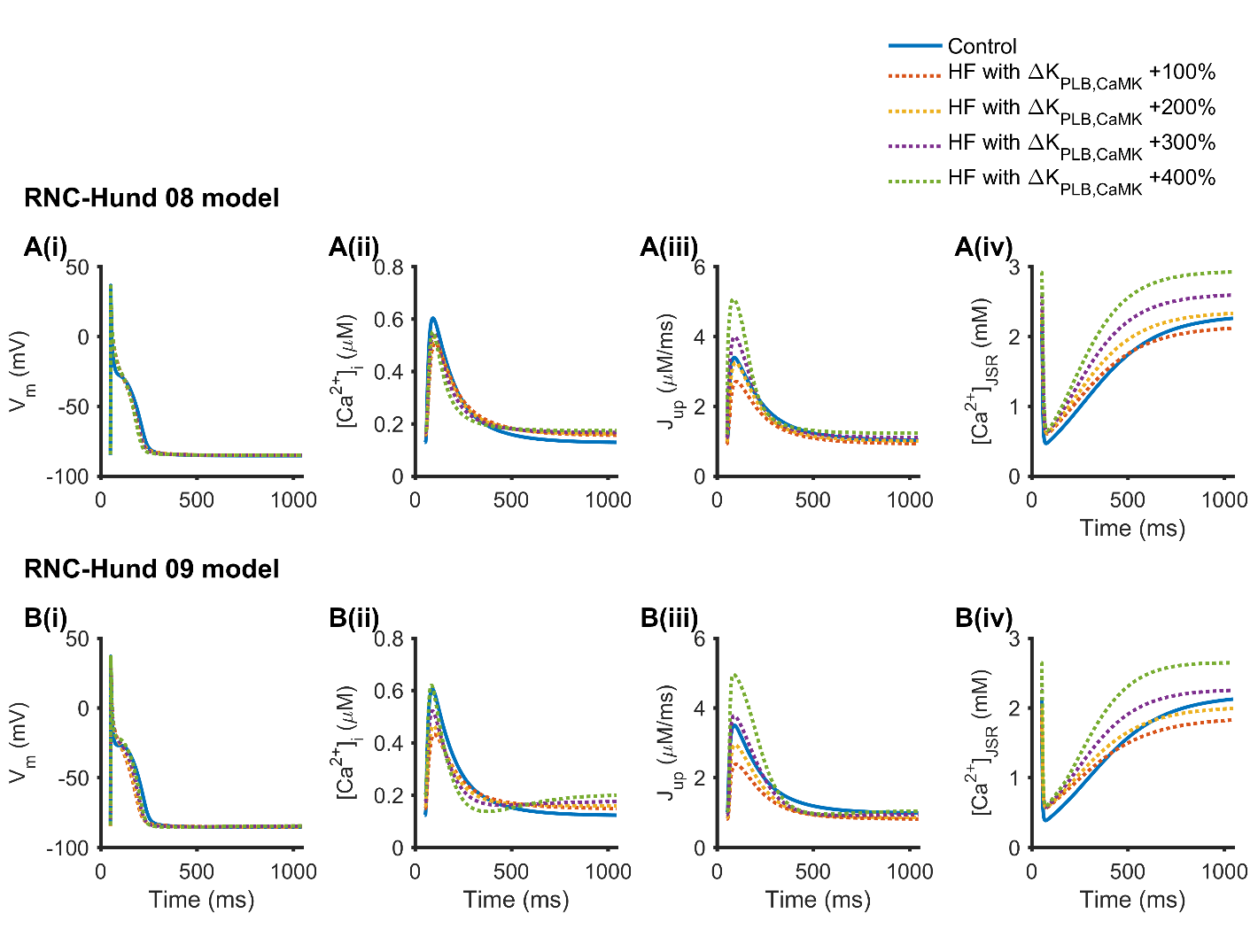


Fig 2. Effect of HF-induced electrical remodelling with the expression of CaMKIIδ isoform increased by 123% and Δ*K*_PLB,CaMK_ increased by 100% to 400% on the AP (i), Ca^2+^ transient (ii), SERCA Ca^2+^ uptake (*J*_up_) (iii) and JSR Ca^2+^ content (iv) in RNC-Hund 08 model (A) and RNC-Hund 09 model (B).

**Reference**

1. Hund TJ, Decker KF, Kanter E, Mohler PJ, Boyden PA, Schuessler RB, Yamada KA, Rudy Y. Role of activated CaMKII in abnormal calcium homeostasis and I(Na) remodeling after myocardial infarction: insights from mathematical modeling. J Mol Cell Cardiol. 2008; 45(3):420-8. doi: 10.1016/j.yjmcc.2008.06.007.
2. Christensen MD, Dun W, Boyden PA, Anderson ME, Mohler PJ, Hund TJ. Oxidized calmodulin kinase II regulates conduction following myocardial infarction: a computational analysis. PLoS Comput Biol. 2009;5(12):e1000583. doi: 10.1371/journal.
3. Ramirez RJ, Nattel S, Courtemanche M. Mathematical analysis of canine atrial action potentials: rate, regional factors, and electrical remodeling. Am J Physiol Heart Circ Physiol. 2000;279(4):H1767-85. doi: 10.1152/ajpheart.2000.279.4.H1767.
4. Li D, Melnyk P, Feng J, Wang Z, Petrecca K, Shrier A, et al. Effects of experimental heart failure on atrial cellular and ionic electrophysiology. Circulation. 2000;101(22):2631-8. doi: 10.1161/01.cir.101.22.2631.
5. Cha TJ, Ehrlich JR, Zhang L, Nattel S. Atrial ionic remodeling induced by atrial tachycardia in the presence of congestive heart failure. Circulation. 2004;110(12):1520-6. doi: 10.1161/01.Cir.0000142052.03565.87.
6. Yeh YH, Wakili R, Qi XY, Chartier D, Boknik P, Kaab S, et al. Calcium-handling abnormalities underlying atrial arrhythmogenesis and contractile dysfunction in dogs with congestive heart failure. Circ Arrhythm Electrophysiol. 2008;1(2):93-102. doi: 10.1161/circep.107.754788.
7. Yoo S, Aistrup G, Shiferaw Y, Ng J, Mohler PJ, Hund TJ, et al. Oxidative stress creates a unique, CaMKII-mediated substrate for atrial fibrillation in heart failure. JCI Insight. 2018;3(21). pii: 120728. doi: 10.1172/jci.insight.120728.
8. Tessier S, Karczewski P, Krause EG, Pansard Y, Acar C, Lang-Lazdunski M, et al. Regulation of the transient outward K(+) current by Ca(2+)/calmodulin-dependent protein kinases II in human atrial myocytes. Circ Res. 1999;85(9):810–819.
9. Wagner S, Hacker E, Grandi E, Weber SL, Dybkova N, Sossalla S, et al. Ca/calmodulin kinase II differentially modulates potassium currents. Circ Arrhythm Electrophysiol. 2009;2(3):285-94. doi: 10.1161/CIRCEP.108.842799.
